# Causal Association of Circulating Adipokines With the Risk of Carpal Tunnel Syndrome and Diabetic Neuropathy: A Bidirectional Two‐Sample Mendelian Randomization Study

**DOI:** 10.1155/jdr/9935331

**Published:** 2026-03-10

**Authors:** Hongquan Wen, Zhiqiang Fan, Pengfei Wang, Jia Li

**Affiliations:** ^1^ Department of Orthopedic Trauma, Xi′an Honghui Hospital, Xi′an Jiaotong University, Xi′an, Shaanxi, China, xjtu.edu.cn; ^2^ Department of Anesthesiology, Xi′an Honghui Hospital, Xi′an Jiaotong University, Xi′an, Shaanxi, China, xjtu.edu.cn

**Keywords:** adiponectin, carpal tunnel syndrome, diabetic neuropathies, interleukin-6, leptin, Mendelian randomization analysis, resistin

## Abstract

This study is aimed at exploring the causal association between genetically predicted serum levels of circulating adipokines and the risk of carpal tunnel syndrome (CTS) and diabetic neuropathy (DN). A two‐sample bidirectional Mendelian randomization (MR) design was employed, using the inverse‐variance weighted (IVW), weighted median, weighted mode, and MR‐Egger regression methods. Sensitivity tests included MR‐Egger, MR‐PRESSO, Cochran′s *Q*, and leave‐one‐out methods. A total of 52 single‐nucleotide polymorphisms (SNPs) associated with adiponectin, leptin, resistin, and IL‐6 levels were selected as instrumental variables (IVs). With adiponectin as exposure, no statistically significant causal association was confirmed for CTS (OR [95% CI]: 1.0331 [0.8257–1.2925], *p* = 0.776) or DN (OR [95% CI]: 0.865 [0.5385–1.3894], *p* = 0.549). With leptin levels as exposure, no statistically significant causal association was confirmed for CTS (OR [95% CI]: 0.8958 [0.5069–1.583], *p* = 0.705) or DN (OR [95% CI]: 1.6745 [0.6704–4.1828], *p* = 0.270) as well. With IL‐6 levels as exposure, no statistically significant causal association for CTS (OR [95% CI]: 1.0037 [0.9627–1.0465], *p* = 0.861) or DN (OR [95% CI]: 1.0213 [0.9177–1.1366], *p* = 0.699) was found. With resistin levels as exposure, no statistically significant causal association was confirmed for CTS (OR [95% CI]: 0.9867 [0.8452–1.1518], *p* = 0.865) or DN (OR [95% CI]: 0.9751 [0.7358–1.2924], *p* = 0.861). The result of MR‐Egger regression and other sensitivity methods suggested that analyses are robust and not affected by horizontal pleiotropy. This study did not find the conclusive evidence that genetically predicted levels of adiponectin, leptin, resistin, and IL‐6 are causally associated with the risk of CTS or DN. The relationship between circulating adipokines and neuropathies is most likely heavily moderated by confounders, such as obesity and hyperglycemia.

## 1. Introduction

Carpal tunnel syndrome (CTS) is a chronic compression of the median wrist nerve at the level of the flexor retinaculum [1]. With a prevalence of approximately 5% worldwide, CTS is one the most common neuropathies, often accompanying diabetes, hypothyroidism, or obesity and significantly affecting quality of life for many people [1, 2]. Diabetic neuropathy (DN) is the most frequent complication of diabetes, occurring in 23%–54% of diabetic patients [3, 4]. Moreover, diabetes increases the risk for other neuropathic complications, and diabetic patients reportedly have 90% higher odds of developing CTS compared with nondiabetics [5]. Although CTS and DN are common and somewhat expected pathologies, the diagnosis is often delayed due to the lack of effective hematological prediction or diagnostic indicators.

Although several biochemical and structural factors contribute to the CTS in diabetes, the exact relationship remains unclear. One of the recently discovered potential links is the obesity‐driven changes and the development of metabolic syndrome [6, 7]. Adipose tissue is widely recognized as an endocrine organ, secreting various adipokines that act as classic hormones, influencing the metabolism of tissues and organs due to their specific receptors on target cells [8]. Adipokines were previously shown to decrease insulin sensitivity in tissues, induce inflammation, and contribute to the development of diabetes [9, 10]. In a number of experimental and observational studies, adiponectin, the most abundant adipokine, improved DN by inhibiting necrotic apoptosis [7], and showed a potential to inhibit fibrosis in CTS [11]; transferrin levels demonstrated a significant predictive value in the early stages of diabetes [12]; interleukin‐6 (IL‐6) levels were associated with the risk of CTS [13]. However, other authors did not find a consistent relationship between adipokines and neuropathy [14, 15] or observed associations to vary between obese and nonobese individuals [16], pointing out that the causal relationship between adipokines and neuropathy remained unclear.

Mendelian randomization (MR) is a tool to limit the influence of confounding factors and reverse causality by using the genetic variants associated with the exposure to some factors in lieu of real‐life exposure [17]. This approach could model the experimental conditions, such as changes in adipokines, using the data from genome‐wide association studies (GWAS) with the promising degree of validity [18]. In particular, previous MR studies revealed the causal association between genetically predicted levels of resistin and atrial fibrillation [18], or adiponectin, leptin, and resistin with the risk of knee osteoarthritis [19], as well as reverse link between adiponectin levels and the idiopathic pulmonary fibrosis [20]. On the other hand, MR research confirmed obesity and diabetes [10], as well as long‐term hyperglycemia [21] to be independent risk factors of CTS, suggesting the connection between two conditions. Using MR approach to model the levels of common adipokines would help to confirm or disprove the hypothesis that circulating adipokines notably influence the development of DN and CTS, and—as such—could be successfully used as diagnostic or therapeutic targets.

Therefore, this study is aimed at exploring the causal associations between genetically predicted levels of circulating adipokines, including adiponectin, leptin, resistin, and IL‐6, with the risk of CTS and DN.

## 2. Materials and Methods

### 2.1. Study Design

**Figure 1:**
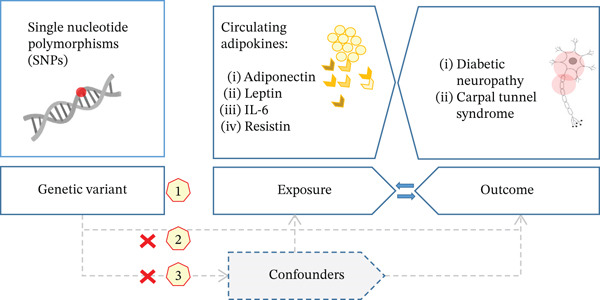
Inclusion and exclusion criteria for instrumental variables (IVs) based on three main principles of avoiding bias in Mendelian randomization study: (1) IVs that significantly associated with changes in selected adipokines were included, (2) IVs should not be directly associated with outcomes, and (3) IVs should not be directly associated with any known confounders.

This was a two‐sample bidirectional MR study that used open data on single‐nucleotide polymorphisms (SNPs) from published GWAS as instrumental variables (IVs). Inclusion and exclusion criteria for IVs were based on three main principles of avoiding bias in MR study [17]: (1) IVs that significantly associated with changes in selected adipokines were included, (2) IVs should not be directly associated with outcomes, and (3) IVs should not be directly associated with any known confounders. The roadmap and hypothesis of the study is demonstrated on Figure 1.

The necessity of informed consent from participants was waived due to the public nature of the data and retrospective approach. The study design strictly followed the MR‐STROBE specifications [22].

### 2.2. Data Source

As exposures, we used four circulating adipokines, adiponectin, leptin, resistin, and IL‐6, with GWAS data for circulating adiponectin levels obtained from the study by Dastani et al. [23] published in 2012 (although the original study included multiethnic participants, for the purposes of this analysis, a separate dataset was used for 29,347 European ancestry individuals). The GWAS data for circulating leptin levels were obtained from the study by Kilpeläinen et al. [24] published in 2016 that included 33,987 European ancestry individuals. The GWAS data for circulating resistin levels were obtained from the study by Folkersen et al. [25] published in 2020 that included 21,758 European ancestry individuals. Finally, data for IL‐6 were obtained from the study by Suhre et al. [26] published in 2017 that included 1000 European ancestry individuals.

Outcome data for CTS, DN, and diabetic polyneuropathy (DPN) were obtained from the FinnGen consortium (R12 release)—a large public–private partnership integrating genome and national health registry data from over 500,000 Finnish biobank participants (http://www.finngen.fi/en). DN in this study refers to generalized neuropathy in diabetes, whereas DPN represents the polyneuropathic subtype; these are analyzed separately for clarity throughout the manuscript.

To evaluate the possibility of reverse causality, a reverse MR analysis was performed, using CTS, DN, and DPN as exposures and circulating adipokines as outcomes.

Detailed identification information and characteristics of all datasets are demonstrated in Table S1.

### 2.3. IV Selection

In this study, IVs were included based on the following criteria:

1. Significance threshold: SNPs associated with each adipokine trait were selected at *p* < 5 × 10^−8^ for adiponectin, *p* < 5 × 10^−6^ for leptin and resistin, and *p* < 5 × 10^−5^ for IL‐6. This gradation was applied because certain GWASs, particularly for leptin, resistin, and IL‐6, contained fewer genome‐wide significant variants at the strict 5 × 10^−8^ threshold. Therefore, slightly relaxed thresholds ensured a sufficient number of instruments for analysis while maintaining acceptable instrument strength. Instrument validity was further confirmed through *F*‐statistic calculations (all *F* > 10). For the reverse MR, SNPs associated with CTS and DN were chosen using genome‐wide significance of *p* < 5 × 10^−6^ (or stricter, when possible), balancing availability and statistical power [27, 28].
2. MAF: Only SNPs with MAF > 0.01 were retained.
3. LD: LD‐clumping applied R^2^ < 0.001 within a 10,000 kb window [22].
4. Proxy SNP substitution: If a selected SNP was absent in the outcome dataset, a proxy with R^2^ > 0.8 was adopted.
5. Instrument strength: The *F*‐statistic for each SNP was calculated using F = R^2^ × (N − 2)/(1 − R^2^), where R^2^ represents the proportion of exposure variance explained by each SNP. Only IVs with *F* > 10 were retained to minimize weak‐instrument bias [22].

### 2.4. MR Analysis

The primary causal inference relied on the inverse‐variance weighted (IVW) method, with effect sizes expressed as odds ratios (OR) and 95% confidence intervals (CIs) [27]. To test robustness under potential violations of MR assumptions, the MR‐Egger, weighted median, and weighted mode estimators were additionally applied. All analyses were performed in R v4.3.0 using the “TwoSampleMR” package, and results were visualized through scatter and forest plots for each adipokine–neuropathy pair.

### 2.5. Sensitivity Analysis

To evaluate heterogeneity, Cochran′s *Q* statistic was used, applying a random‐effects IVW model when heterogeneity (*p* < 0.05) was detected. Directional pleiotropy was assessed using the MR‐Egger intercept (*p* > 0.05 indicating no evidence of pleiotropy). Potential pleiotropic outliers were identified using MR‐PRESSO, and all analyses were repeated after outlier removal when applicable. Finally, leave‐one‐out analyses assessed whether any single IV unduly influenced the effect estimates. These sensitivity analyses were performed for both forward and reverse MR directions. Because potential confounding factors such as body mass index (BMI) and Type 2 diabetes are closely interrelated with adipokine pathways, we considered applying multivariable MR (MVMR) to adjust for them. However, shared valid instruments across exposures and confounders were insufficient, precluding reliable MVMR modelling in this dataset.

## 3. Results

### 3.1. IV Selection

When IVs were selected with adiponectin levels as exposure, 12 SNPs were included and the mean *F*‐statistic was 81.19 (33.00, 314.82); with leptin levels as exposure, 10 SNPs were included, and the mean *F*‐statistic was 24.19 (21.21, 28.95); with resistin levels as exposure, 13 SNPs were included, and the mean *F*‐statistic was 64.96 (30.07, 192.11); with IL‐6 as exposure, 17 SNPs were included, and the mean *F*‐statistic was 18.41 (16.68, 20.81). All SNPs used, including those excluded or replaced, are listed in Table S2.

In the reverse MR for CTS as exposure, 54 SNPs were used (mean *F* − statistic = 23.96); for DN as exposure, seven SNPs were used (mean *F* − statistic = 68.73); and for DPN as exposure, nine SNPs were used (mean *F* − statistic = 22.29). Proxy SNPs were applied where original variants could not be matched, and weak or palindromic instruments were excluded where appropriate.

Full details of the SNPs used as IVs are provided in Table S2.

### 3.2. MR Analysis Results

When MR analysis was performed with adiponectin levels as exposure (Figures 2a, 2b, 2c, 2d, 2e, and 2f), no statistically significant causal association was confirmed for CTS (OR = 1.0331, 95% CI: 0.8257–1.2925, *p* = 0.776), DN (OR = 0.865, 95% CI: 0.5385–1.3894, *p* = 0.549), or DPN (OR = 0.8197, 95% CI: 0.3272–2.054, *p* = 0.672). When MR analysis was performed with leptin levels as exposure (Figures 3a, 3b, 3c, 3d, 3e, and 3f), no statistically significant causal association was confirmed for CTS (OR = 0.8958, 95% CI: 0.5069–1.583, *p* = 0.705), DN (OR = 1.6745, 95% CI: 0.6704–4.1828, *p* = 0.270), or DPN (OR = 1.4689, 95% CI: 0.2243–9.6204, *p* = 0.688) as well. When MR analysis was performed with IL‐6 levels as exposure (Figures 4a, 4b, 4c, 4d, 4e, and 4f), no statistically significant causal association for CTS (OR = 1.0037, 95% CI: 0.9627–1.0465, *p* = 0.861), DN (OR = 1.0213, 95% CI: 0.9177–1.1366, *p* = 0.699), or DPN (OR = 1.136, 95% CI: 0.9452–1.3654, *p* = 0.174) was found. And finally, when MR analysis was performed with resistin levels as exposure (Figures 5a, 5b, 5c, 5d, 5e, and 5f), no statistically significant causal association was confirmed for CTS (OR = 0.9867, 95% CI: 0.8452–1.1518, *p* = 0.865), DN (OR = 0.9751, 95% CI: 0.7358–1.2924, *p* = 0.861), or DPN (OR = 1.0387, 95% CI: 0.569–1.8961, *p* = 0.902).

Figure 2Visualization of associations between adiponectin plasma levels and carpal tunnel syndrome (a), diabetic neuropathy (b), and diabetic polyneuropathy (c); results of leave‐one‐out method suggesting that results are not driven by any single factor, demonstrated for adiponectin plasma levels and carpal tunnel syndrome (d), diabetic neuropathy (e), and diabetic polyneuropathy (f) through scatter and forest plots.

**Figure:**
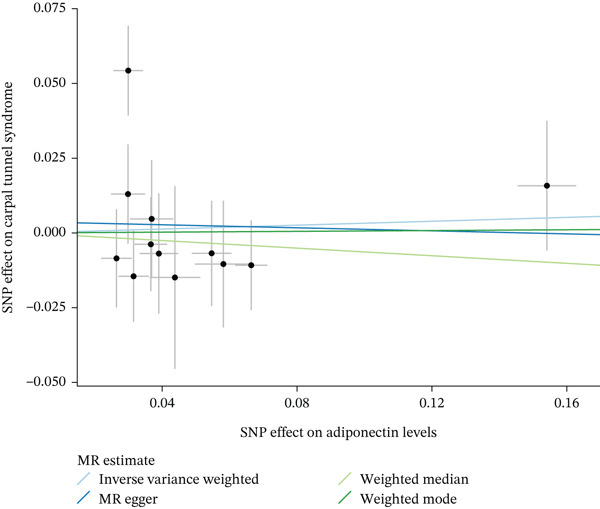
(a)

**Figure:**
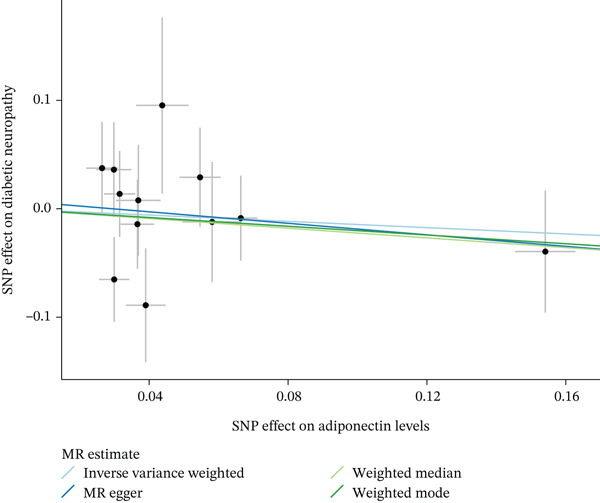
(b)

**Figure:**
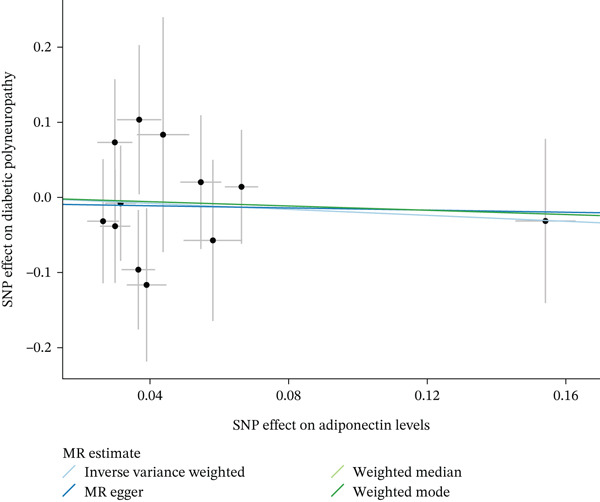
(c)

**Figure:**
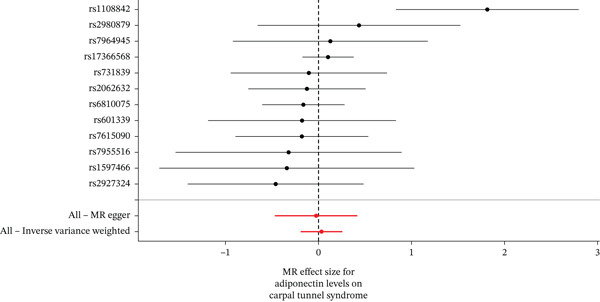
(d)

**Figure:**
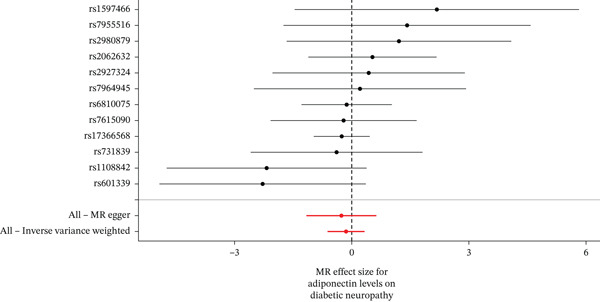
(e)

**Figure:**
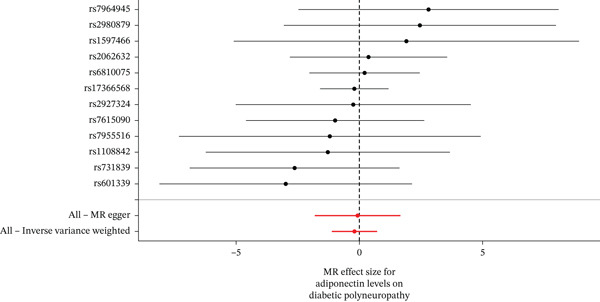
(f)

Figure 3Visualization of associations between leptin plasma levels and carpal tunnel syndrome (a), diabetic neuropathy (b), diabetic polyneuropathy (c); results of leave‐one‐out method suggesting that results are not driven by any single factor, demonstrated for leptin plasma levels and carpal tunnel syndrome (d), diabetic neuropathy (e), and diabetic polyneuropathy (f) through scatter and forest plots.

**Figure:**
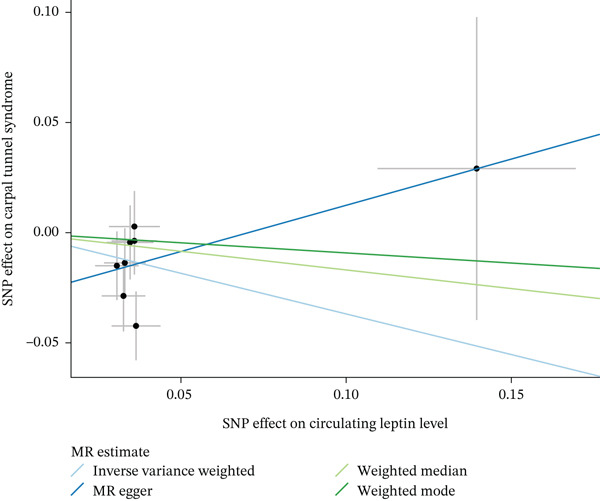
(a)

**Figure:**
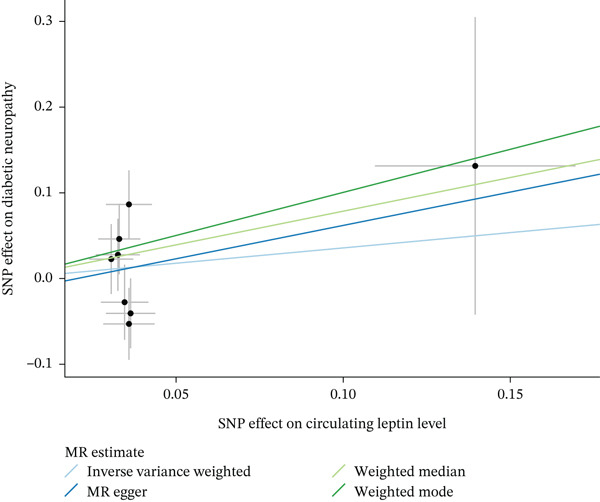
(b)

**Figure:**
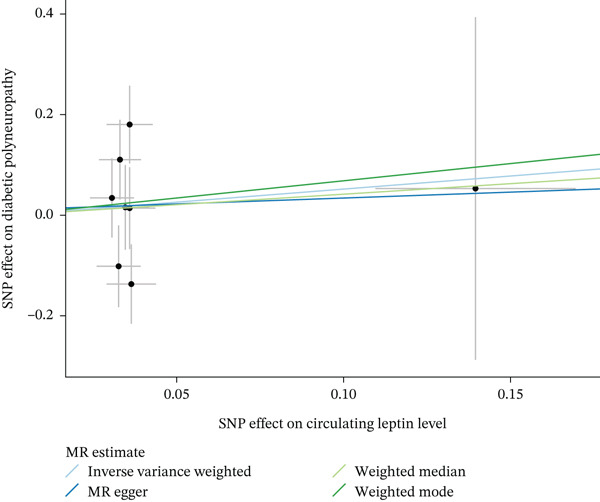
(c)

**Figure:**
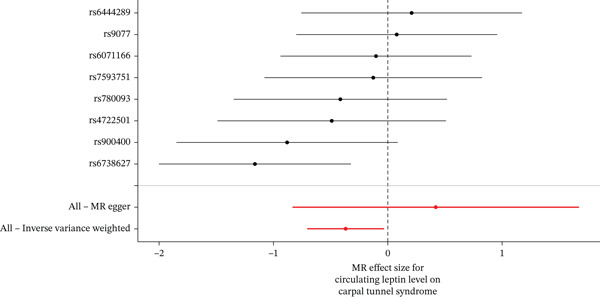
(d)

**Figure:**
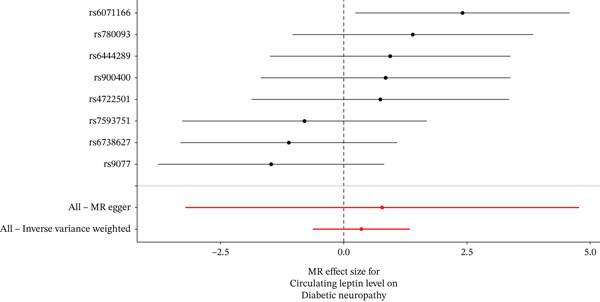
(e)

**Figure:**
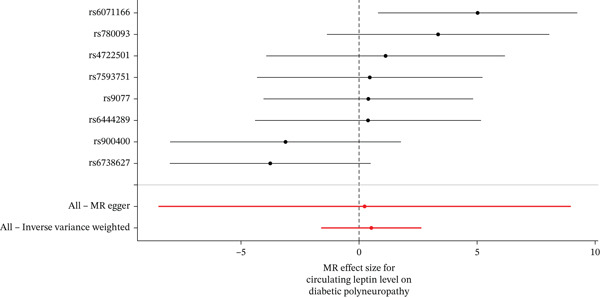
(f)

Figure 4Visualization of associations between IL‐6 plasma levels and carpal tunnel syndrome (a), diabetic neuropathy (b), diabetic polyneuropathy (c); results of leave‐one‐out method suggesting that results are not driven by any single factor, demonstrated for IL‐6 plasma levels and carpal tunnel syndrome (d), diabetic neuropathy (e), and diabetic polyneuropathy (f) through scatter and forest plots.

**Figure:**
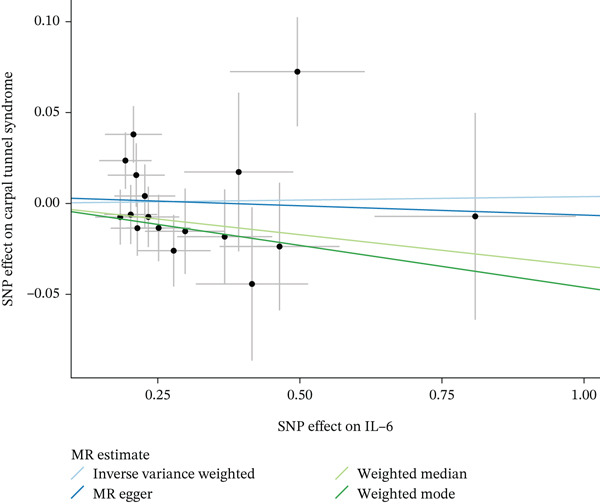
(a)

**Figure:**
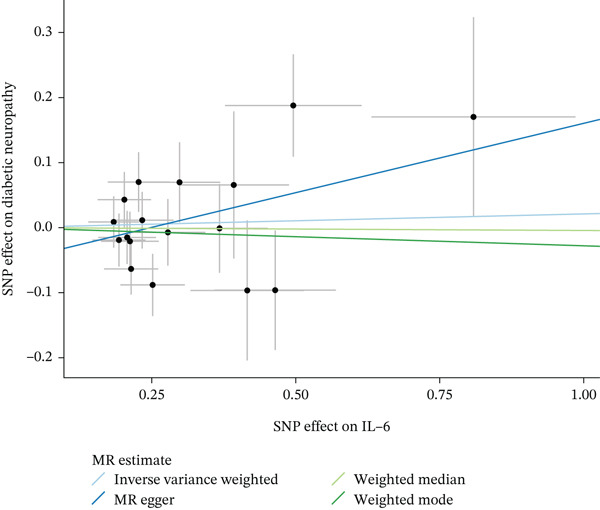
(b)

**Figure:**
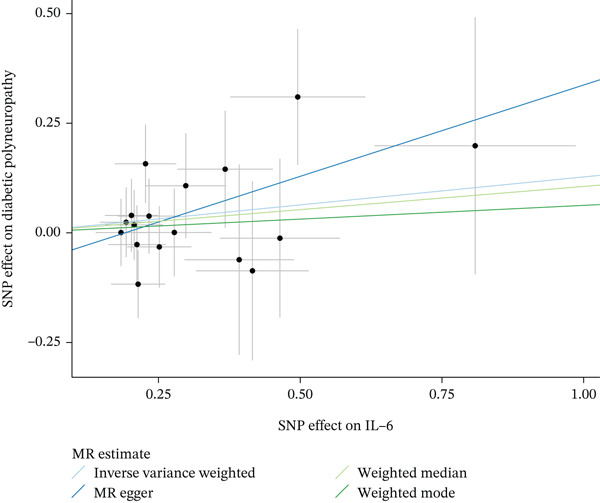
(c)

**Figure:**
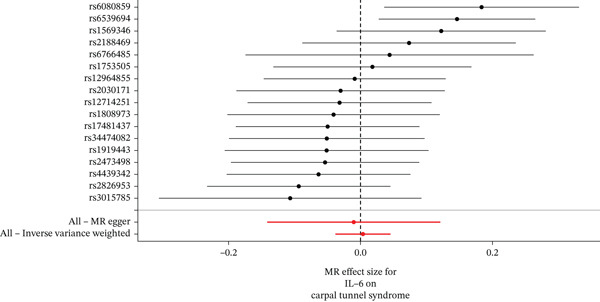
(d)

**Figure:**
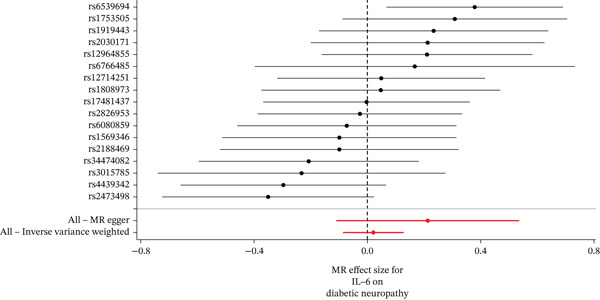
(e)

**Figure:**
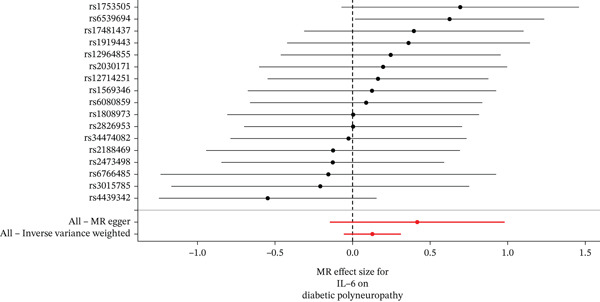
(f)

Figure 5Visualization of associations between resistin plasma levels and carpal tunnel syndrome (a), diabetic neuropathy (b), diabetic polyneuropathy (c); results of leave‐one‐out method suggesting that results are not driven by any single factor, demonstrated for resistin plasma levels and carpal tunnel syndrome (d), diabetic neuropathy (e), and diabetic polyneuropathy (f) through scatter and forest plots.

**Figure:**
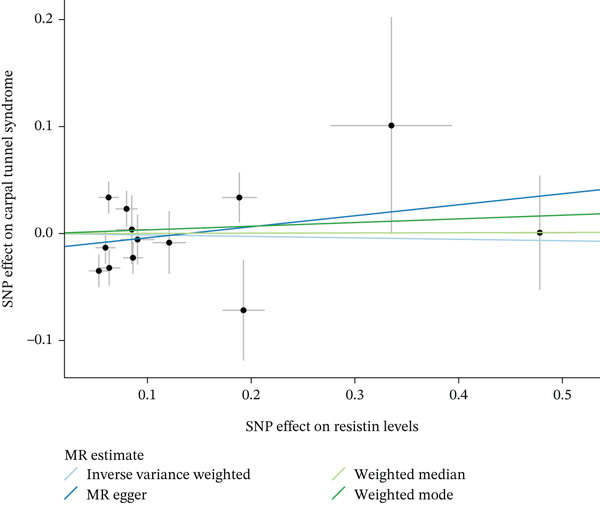
(a)

**Figure:**
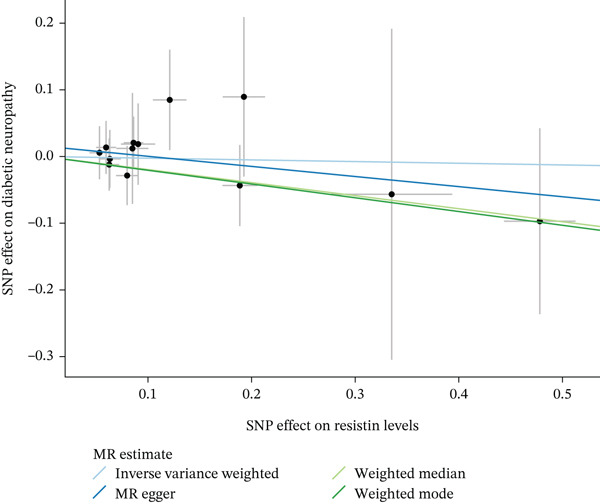
(b)

**Figure:**
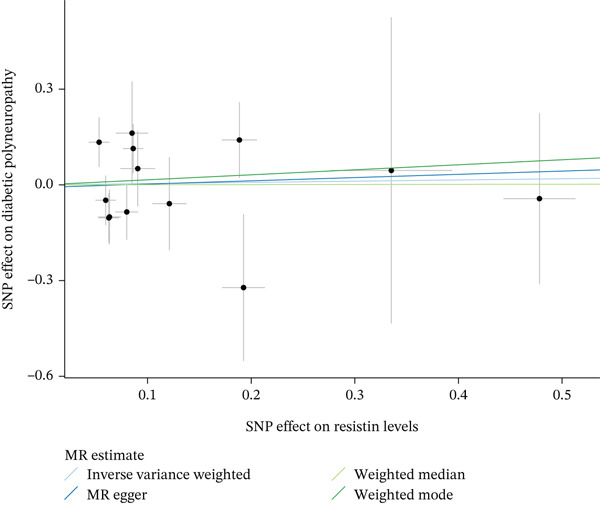
(c)

**Figure:**
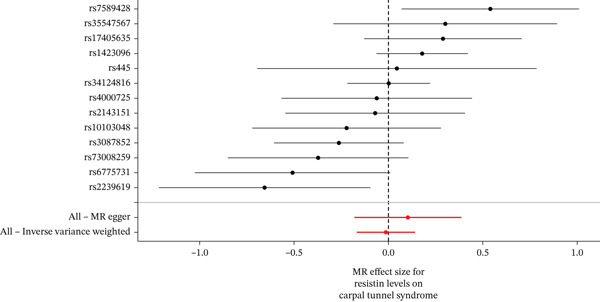
(d)

**Figure:**
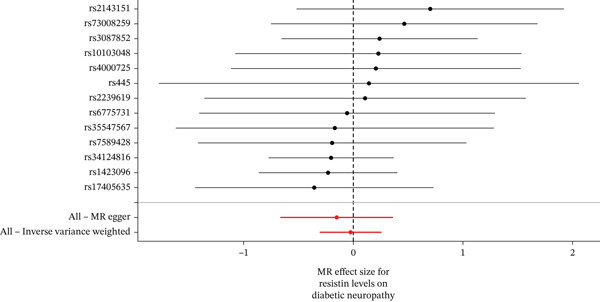
(e)

**Figure:**
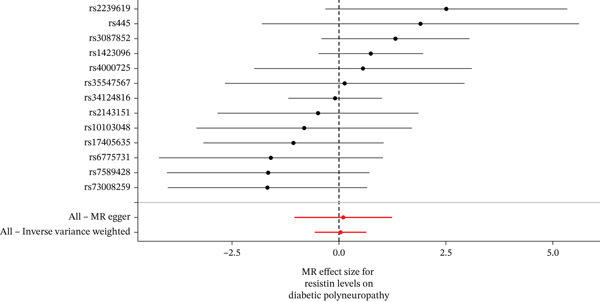
(f)

Application of other MR methods confirmed the absence of significant associations, as demonstrated in Table 1.

**Table 1:** The association between circulating adipokine levels and the risk of carpal tunnel syndrome and diabetic neuropathy.

**Outcome**	**Exposure**	**Methods**	**No**	**p**	**OR (95% CI)**
Carpal tunnel syndrome	Circulating leptin level	IVW	9	0.705	0.8958 (0.5069–1.583)
	Circulating leptin level	MR‐Egger	9	0.821	1.3473 (0.1118–16.2339)
	Circulating leptin level	Weighted median	9	0.583	0.8849 (0.5716–1.3698)
	Circulating leptin level	Weighted mode	9	0.734	0.8961 (0.4871–1.6488)
Carpal tunnel syndrome	Resistin levels	IVW	13	0.865	0.9867 (0.8452–1.1518)
	Resistin levels	MR‐Egger	13	0.492	1.1083 (0.8348–1.4714)
	Resistin levels	Weighted median	13	0.982	1.002 (0.8476–1.1845)
	Resistin levels	Weighted mode	13	0.742	1.0349 (0.8479–1.2632)
Carpal tunnel syndrome	Adiponectin levels	IVW	12	0.776	1.0331 (0.8257–1.2925)
	Adiponectin levels	MR‐Egger	12	0.913	0.9749 (0.6259–1.5185)
	Adiponectin levels	Weighted median	12	0.595	0.9385 (0.7428–1.1857)
	Adiponectin levels	Weighted mode	12	0.960	1.0067 (0.7812–1.2972)
Carpal tunnel syndrome	IL‐6	IVW	17	0.861	1.0037 (0.9627–1.0465)
	IL‐6	MR‐Egger	17	0.880	0.9898 (0.8681–1.1286)
	IL‐6	Weighted median	17	0.202	0.9662 (0.9165–1.0186)
	IL‐6	Weighted mode	17	0.293	0.9549 (0.8786–1.0378)
Diabetic neuropathy	Circulating leptin level	IVW	9	0.270	1.6745 (0.6704–4.1828)
	Circulating leptin level	MR‐Egger	9	0.735	2.0229 (0.0401–101.9841)
	Circulating leptin level	Weighted median	9	0.154	2.3397 (0.7274–7.5256)
	Circulating leptin level	Weighted mode	9	0.330	3.3196 (0.3437–32.0629)
Diabetic neuropathy	Resistin levels	IVW	13	0.861	0.9751 (0.7358–1.2924)
	Resistin levels	MR‐Egger	13	0.575	0.8594 (0.5143–1.436)
	Resistin levels	Weighted median	13	0.314	0.8223 (0.5621–1.2031)
	Resistin levels	Weighted mode	13	0.394	0.8137 (0.5153–1.285)
Diabetic neuropathy	Adiponectin levels	IVW	12	0.549	0.865 (0.5385–1.3894)
	Adiponectin levels	MR‐Egger	12	0.574	0.7669 (0.3132–1.8782)
	Adiponectin levels	Weighted median	12	0.462	0.7995 (0.4406–1.4506)
	Adiponectin levels	Weighted mode	12	0.531	0.8174 (0.4434–1.5067)
Diabetic neuropathy	IL‐6	IVW	17	0.699	1.0213 (0.9177–1.1366)
	IL‐6	MR‐Egger	17	0.215	1.2376 (0.896–1.7095)
	IL‐6	Weighted median	17	0.949	0.9956 (0.8708–1.1383)
	IL‐6	Weighted mode	17	0.837	0.9724 (0.748–1.2642)
Diabetic polyneuropathy	Circulating leptin level	IVW	9	0.688	1.4689 (0.2243–9.6204)
	Circulating leptin level	MR‐Egger	9	0.945	1.3461 (4e‐04–4687.0069)
	Circulating leptin level	Weighted median	9	0.735	1.4789 (0.1529–14.3033)
	Circulating leptin level	Weighted mode	9	0.841	1.4489 (0.0431–48.6819)
Diabetic polyneuropathy	Resistin levels	IVW	13	0.902	1.0387 (0.569–1.8961)
	Resistin levels	MR‐Egger	13	0.865	1.1071 (0.3533–3.4688)
	Resistin levels	Weighted median	13	0.993	1.0034 (0.4579–2.1988)
	Resistin levels	Weighted mode	13	0.753	1.1699 (0.4502–3.0402)
Diabetic polyneuropathy	Adiponectin levels	IVW	12	0.672	0.8197 (0.3272–2.054)
	Adiponectin levels	MR‐Egger	12	0.937	0.9309 (0.1639–5.289)
	Adiponectin levels	Weighted median	12	0.802	0.8677 (0.2868–2.6251)
	Adiponectin levels	Weighted mode	12	0.826	0.8673 (0.2509–2.9974)
Diabetic polyneuropathy	IL‐6	IVW	17	0.174	1.136 (0.9452–1.3654)
	IL‐6	MR‐Egger	17	0.168	1.517 (0.8637–2.6647)
	IL‐6	Weighted median	17	0.410	1.1117 (0.864–1.4303)
	IL‐6	Weighted mode	17	0.794	1.0647 (0.67–1.6918)
After removing the outliers					

Exposure	Outcome	Method	No. of SNPs	*p*	OR (95% CI)

Circulating leptin level	Carpal tunnel syndrome	Inverse‐variance weighted	7	0.198	0.7943 (0.5592–1.1281)
Circulating leptin level	Carpal tunnel syndrome	Inverse‐variance weighted	7	0.198	0.7943 (0.5592–1.1281)
Circulating leptin level	Carpal tunnel syndrome	MR‐Egger	7	0.47	1.6492 (0.4698–5.7887)
Circulating leptin level	Carpal tunnel syndrome	Weighted median	7	0.598	0.8870 (0.5679–1.3855)
Circulating leptin level	Carpal tunnel syndrome	Weighted mode	7	0.828	0.9287 (0.4903–1.7589)
Resistin levels	Carpal tunnel syndrome	Inverse‐variance weighted	11	0.176	0.9005 (0.7736–1.0482)
Resistin levels	Carpal tunnel syndrome	MR‐Egger	11	0.565	1.0790 (0.8408–1.3846)
Resistin levels	Carpal tunnel syndrome	Weighted median	11	0.571	0.9498 (0.7950–1.1348)
Resistin levels	Carpal tunnel syndrome	Weighted mode	11	0.659	0.9528 (0.7732–1.1740)

In the reverse MR analysis, no evidence of reverse causality was found between DN, DPN, CTS and circulating levels of adiponectin, leptin, resistin, or IL‐6. All IVW *p* values were > 0.05, and the directionality of effects was inconsistent across outcomes (Table S3).

### 3.3. Results of Sensitivity Analysis

The results of MR‐Egger regression suggested that our analyses were largely unaffected by directional pleiotropy, as demonstrated in Table 2 and Table S4. However, significant heterogeneity was detected for the associations of circulating leptin (Cochran^′^s *Q* *p* < 0.001) and resistin (Cochran^′^s *Q* *p* = 0.018) with CTS (Table 2).

**Table 2:** Results of heterogeneity test and pleiotropy test of instrumental variables.

**Outcome**	**Exposure**	**Hetergeneity**		**Pleiotropy**	
		**Q**	**p**	**Egger_intercept**	**p**

Carpal tunnel syndrome	Circulating leptin level	28.102	≤ 0.001	−0.015	0.750
	Resistin levels	24.395	0.018	−0.014	0.357
	Adiponectin levels	16.683	0.118	0.004	0.769
	IL‐6	21.190	0.171	0.004	0.828
Diabetic neuropathy	Circulating leptin level	10.726	0.218	−0.007	0.925
	Resistin levels	3.894	0.985	0.015	0.576
	Adiponectin levels	9.362	0.588	0.008	0.763
	IL‐6	20.252	0.209	−0.053	0.236
Diabetic polyneuropathy	Circulating leptin level	12.054	0.149	0.003	0.983
	Resistin levels	14.734	0.256	−0.008	0.898
	Adiponectin levels	5.587	0.899	−0.008	0.869
	IL‐6	11.213	0.796	−0.080	0.304
After excluding the outliers					

**Outcome**	**Exposure**	**Heterogeneity**		**Pleiotropy**	
		**Q**	**Egger_intercept**	**Egger_intercept**	**p**

Carpal tunnel syndrome	Circulating leptin level	3.544560029	0.73803205	−0.027491965	0.288255702
Carpal tunnel syndrome	Resistin levels	14.61233675	0.146847476	−0.021506186	0.121023142

To address this, we performed MR‐PRESSO analysis, which identified outlier SNPs for these two associations (Table 3). After removing the identified outliers, we reperformed all sensitivity analyses. For the leptin–CTS association, the heterogeneity was resolved (Cochran^′^s Q *p* = 0.738), whereas for the resistin–CTS association, heterogeneity persisted (Cochran^′^s Q *p* = 0.147). Crucially, for both associations, the causal estimates remained consistently null across all MR methods after outlier removal, confirming the robustness of our primary findings (see Figures S1a, S1b, S1c, S1d, S1e, S1f, S1g, S1h, S1i, S1j, and S1l and Table 1).

**Table 3:** Results of MR‐PRESSO analysis.

**Exposure**	**Outcome**	**RAW**		**Outlier corrected**			**Global** **p**	**Number of outliers**	**Distortion** **p**
		**or_ci**	**p**	**or_ci**		**p**			

Adiponectin levels	Carpal tunnel syndrome	1.03 (0.83 –1.29)	0.781	/		/	0.194	/	/
Circulating leptin level		0.91 (0.54–1.51)	0.717	0.82 (0.64–1.04)		0.149	< 0.001	2 (rs6738627 rs8043757)	0.568
IL‐6		1.00 (0.96 –1.05)	0.863	/		/	0.160	/	/
Resistin levels		0.99 (0.85 –1.15)	0.868	/		/	0.021	/	/
Adiponectin levels	Diabetic neuropathy	0.86 (0.56 –1.34)	0.529	/		/	0.666	/	/
Circulating leptin level		1.81 (0.78 –4.18)	0.200	/		/	0.267	/	/
IL‐6		1.02 (0.92 –1.14)	0.704	/		/	0.193	/	/
Resistin levels		0.98 (0.83 –1.14)	0.764	/		/	0.983	/	/
Adiponectin levels	Diabetic polyneuropathy	0.82 (0.43 –1.58)	0.564	/		/	0.918	/	/
Circulating leptin level		1.48 (0.27 –8.05)	0.658	/		/	0.215	/	/
IL‐6		1.14 (0.97 –1.33)	0.124	/		/	0.789	/	/
Resistin levels		1.04 (0.57 –1.90)	0.904	/		/	0.289	/	/
After excluding the outliers									

**Exposure**	**Outcome**	**RAW**		**Outlier corrected**		**Global** **p**		**Number of outliers**	**Distortion** **p**
		**or_ci**	**p**	**or_ci**	**p**				

Circulating leptin level	Carpal tunnel syndrome	0.79 (0.61–1.04)	0.15	NA (NA–NA)	/	0.748		/	/
Resistin levels	Carpal tunnel syndrome	0.90 (0.77–1.05)	0.21	NA (NA–NA)	/	0.194		/	/

In the reverse MR analysis, a similar outlier‐correction procedure also confirmed the robustness of the null findings where significant heterogeneity was present (Tables S4 and S5). The leave‐one‐out analyses demonstrated that our primary findings were not driven by any single influential SNP (Figures S2a, S2b, S2c, S2d, S2d, S2e, S2f, S2g, S2h, S2i, S2j, S2k, and S2l).

## 4. Discussion

This study employed a bidirectional MR approach to evaluate whether genetically predicted serum levels of circulating adipokines are causally associated with the risk of CTS or DN. Across four major adipokines—adiponectin, leptin, resistin, and IL‐6—no conclusive evidence of causal association was observed. Consistently, the reverse MR analyses provided no support for an effect of CTS, DN, or DPN on circulating adipokine levels. These null findings remained robust across multiple MR estimators and after extensive sensitivity testing, including outlier correction. Despite the important roles of adipokines reported in other metabolic and inflammatory diseases, our results do not support adiponectin, leptin, resistin, or IL‐6 as causal or diagnostic biomarkers for neuropathies.

Adipokines have been widely implicated in inflammatory, fibrotic, and metabolic pathways [7, 11]. One strength of the two‐sample MR design is its capacity to test potential causal relationships independent of confounding and reverse causation. In this study, we sought to clarify whether circulating adipokines alone influence neuropathy risk. Our findings align with prior MR evidence questioning causal adipokine roles in hyperuricemia and gout [29] and idiopathic pulmonary fibrosis [30], but contrast with observational data such as those from Sun et al. [31], who reported a positive association between adiponectin and diabetic peripheral neuropathy. Conversely, our results are consistent with Mohanraj et al. [15], who also found no predictive value of adiponectin for DN. Even after detecting and correcting for heterogeneity and outliers in some reverse‐direction associations, the absence of significant causal effects persisted, reinforcing the robustness of these conclusions. Thus, although circulating adipokines may correlate with neuropathy severity in observational research, these relationships likely reflect downstream or compensatory mechanisms rather than causative effects.

Potential sources of heterogeneity merit brief consideration. Significant heterogeneity for leptin–CTS and resistin–CTS associations was no longer observed after MR‐PRESSO outlier removal, indicating that a few pleiotropic variants disproportionately influenced those results. Biologically, leptin‐related variants may exert broader metabolic effects, including on body‐mass regulation and inflammatory cytokine pathways, whereas resistin variants may participate in immune‐cell activation and extracellular matrix remodeling. These pleiotropic effects could plausibly contribute to context‐specific heterogeneity without representing genuine causal relationships. Importantly, after excluding these outliers, all MR estimates remained null, underscoring the stability of the core findings.

Among possible confounders, obesity remains the most likely mediator linking adipokines, inflammation, and neuropathy. Previous evidence indicates that obesity markedly increases the risks of both DN and CTS [10]. Adipokines are generally considered protective against inflammation and insulin resistance, yet concentrations of adiponectin, leptin, and IL‐4 are often lower in obese individuals [32, 33]. In metabolic syndrome, adiponectin regulation in response to vascular damage differs between obese and nonobese populations, suggesting impaired adipokine modulation with excess adiposity [33]. By contrast, in CTS, adiponectin expression in local adipose tissue surrounding the palmar aponeurosis has been found to increase, possibly reflecting a compensatory antifibrotic response [11]. In our MR framework—where obesity effects were largely adjusted out through the genetic randomization process—these confounding influences were minimized, and no strong causal links were observed. This supports the interpretation that previously noted adipokine changes are secondary responses to disease development rather than initiating factors.

Diabetes itself is another essential contextual factor. It has been associated with higher risks of both DN and CTS [9, 34], leading to speculation that diabetes‐related cytokine shifts could mediate neuropathy. However, the findings across epidemiological studies have been inconsistent. Naha et al. [4] reported that CTS incidence, although higher among individuals with diabetes, was paradoxically lower in those with DN, implying distinct underlying mechanisms. Similarly, a large US cross‐sectional analysis including 322,092 participants [35] did not detect a significant association between diabetes and CTS. Moreover, neuropathic nerves already affected by DN may be more sensitive to compressive forces [36], increasing CTS symptom detection without necessarily affecting incidence. Our MR results, therefore, likely capture this complex interplay: Neither adipokines nor diabetes‐related cytokine alterations appear to have a direct causal role in CTS pathogenesis when genetic confounding is controlled.

This study relied on the strengths of two‐sample MR design, which allows to closely inspect associations between each factor and possible outcome; analyzed data were obtained from different sources with sufficient statistical power, and extended efforts were made to exclude potential horizontal pleiotropy. Additionally, reverse MR analyses were performed to exclude the possibility of reverse causation, which further supports the robustness of our causal inference. However, this study also has several limitations. First, although the two‐sample MR approach models the effects of genetically predicted lifelong exposure to a single adipokine, this estimation inevitably simplifies the complex and interactive biological network of adipokine signaling observed in vivo. Nevertheless, this design provides a valuable perspective on the potential causal direction between adipokines and neuropathic outcomes. Second, some outcomes—particularly DPN—were represented by relatively small case numbers (e.g., approximately 358 cases in FinnGen), which may have limited statistical power and partly contributed to the absence of significant findings. Future studies with larger case counts or meta‐analyses combining multiple cohorts would help to strengthen causal inference. Third, in the reverse MR analyses, the number of valid IVs for several outcomes (e.g., seven SNPs for DN and nine for DPN) was relatively small. Consequently, these reverse MR results should be interpreted with caution, as limited instrument availability may reduce precision and robustness. Fourth, although results of sensitivity analyses were consistent with the IVW estimates and the MR‐Egger regression suggested that pleiotropy was unlikely to affect the findings, the existence of undetected pleiotropy cannot be entirely ruled out. Fifth, we considered applying MVMR to adjust for major confounders such as obesity and Type 2 diabetes; however, the number of shared genetic instruments across adipokines, confounders, and outcomes was insufficient, preventing the construction of stable MVMR models. Finally, this study included only individuals of European ancestry. Furthermore, participants were not stratified by gender or age, even though CTS is more prevalent among females and older populations. Thus, future research in larger, ethnically, and demographically diverse datasets will be valuable for verifying the generalizability of our conclusions.

In conclusion, this study did not find the conclusive evidence that genetically predicted levels of circulating adipokines, including adiponectin, leptin, resistin, and IL‐6, are causally associated with the risk of CTS or DN. The relationship between circulating adipokines and neuropathies is most likely heavily moderated by confounders, such as obesity and hyperglycemia.

## Author Contributions

Conception and design: Hongquan Wen and Jia Li; administrative support: Pengfei Wang; collection and assembly of data: Hongquan Wen and Zhiqiang Fan; data analysis and interpretation: Hongquan Wen and Jia Li; manuscript writing: Hongquan Wen and Jia Li.

## Funding

This study was supported by the National Natural Science Foundation of China (10.13039/501100001809) (82204359) and Natural Science Foundation of Shaanxi Province (2023‐JC‐QN‐0989).

## Disclosure

All authors approved the final manuscript.

## Ethics Statement

The authors have nothing to report.

## Consent

The authors have nothing to report.

## Conflicts of Interest

The authors declare no conflicts of interests.

## Supporting Information

Additional supporting information can be found online in the Supporting Information section.

## Supporting information


**Supporting Information 1** Table S1: Identification information and characteristics of all datasets obtained from genome‐wide association studies (GWAS) for the purposes of this Mendelian randomization analysis. Table S2: List of genetic variants used as instrumental variables in the Mendelian randomization analysis for each of circulating adipokines and neuropathic conditions. Table S3: Mendelian randomization results assessing the association of carpal tunnel syndrome and diabetic neuropathy with circulating adipokine levels. Table S4: Results of heterogeneity test and pleiotropy test of instrumental variables for reverse analysis. Table S5: Application of Mendelian randomization methods after exclusion of outliers (MR‐PRESSO).

**Supporting Information 2** Figure S1: Funnel plots visualizing the heterogeneity of the significant causal associations identified in the main analysis. Figure S2: Leave‐one‐out sensitivity analysis plots for the significant causal associations.

## Data Availability

All data generated or analyzed during this study are included in this published article and its supporting information.
