# Supplementary figures and images for: Causal Association of Circulating Adipokines With the Risk of Carpal Tunnel Syndrome and Diabetic Neuropathy: A Bidirectional Two‐Sample Mendelian Randomization Study
Source: J Diabetes Res. 2026 Mar 10;2026:9935331. doi: 10.1155/jdr/9935331 (PMC12973331; doi:10.1155/jdr/9935331)

# MR Method

Inverse variance weighted

MR Egger

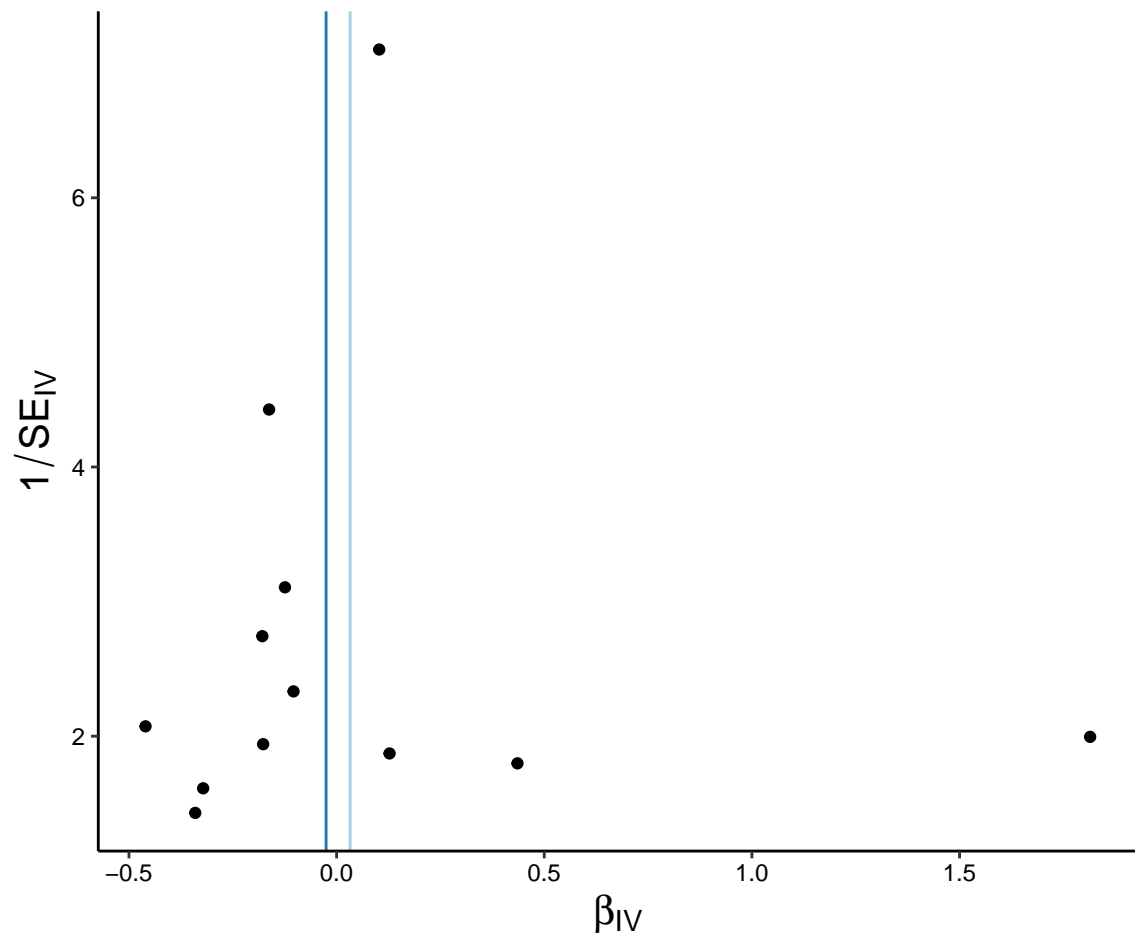

Supplement: Supplementary file 2 — Supporting Information 2 Figure S1: Funnel plots visualizing the heterogeneity of the significant causal associations identified in the main analysis. Figure S2: Leave‐one‐out sensitivity analysis plots for the significant causal associations. [file JDR-2026-9935331-s002.zip › Figure S1/Fig.S1A.pdf]

# MR Method

Inverse variance weighted

MR Egger

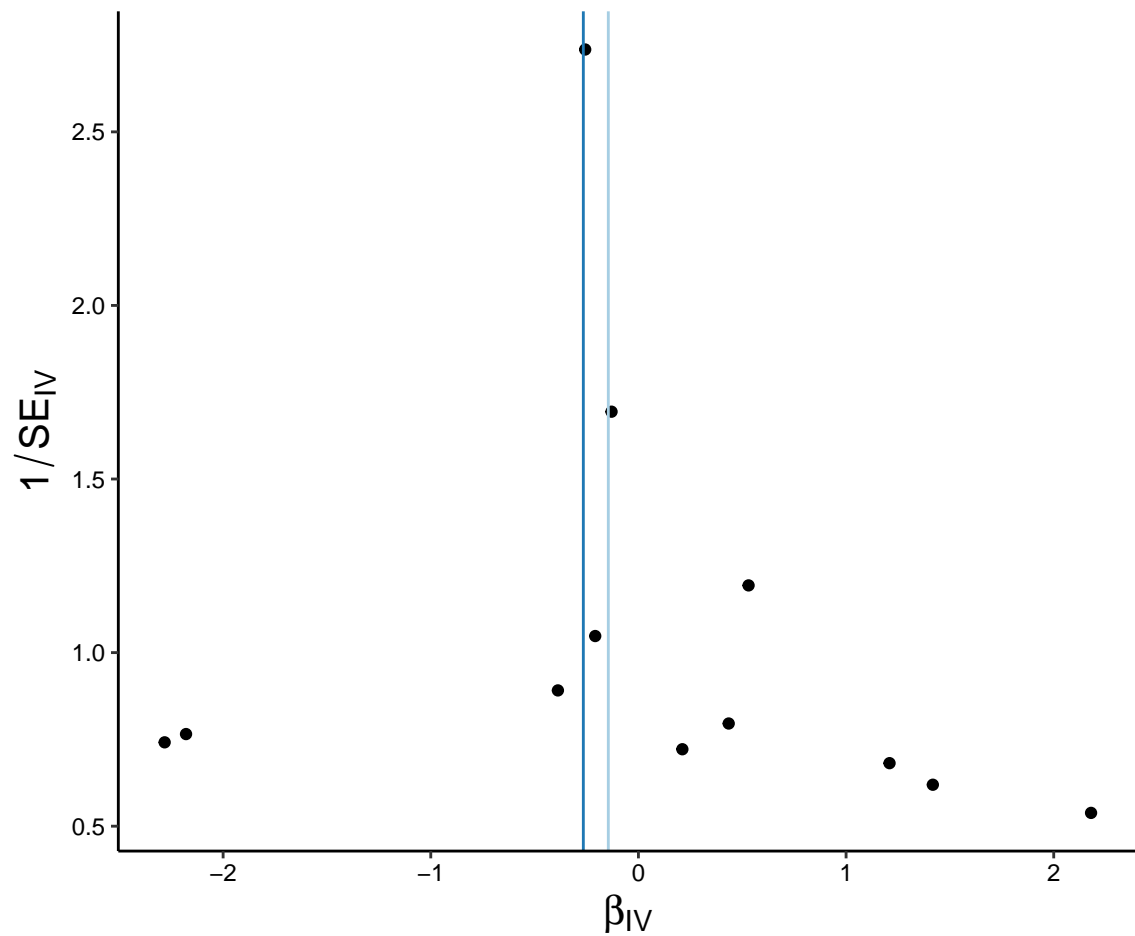

Supplement: Supplementary file 2 — Supporting Information 2 Figure S1: Funnel plots visualizing the heterogeneity of the significant causal associations identified in the main analysis. Figure S2: Leave‐one‐out sensitivity analysis plots for the significant causal associations. [file JDR-2026-9935331-s002.zip › Figure S1/Fig.S1B.pdf]

# MR Method

Inverse variance weighted

MR Egger

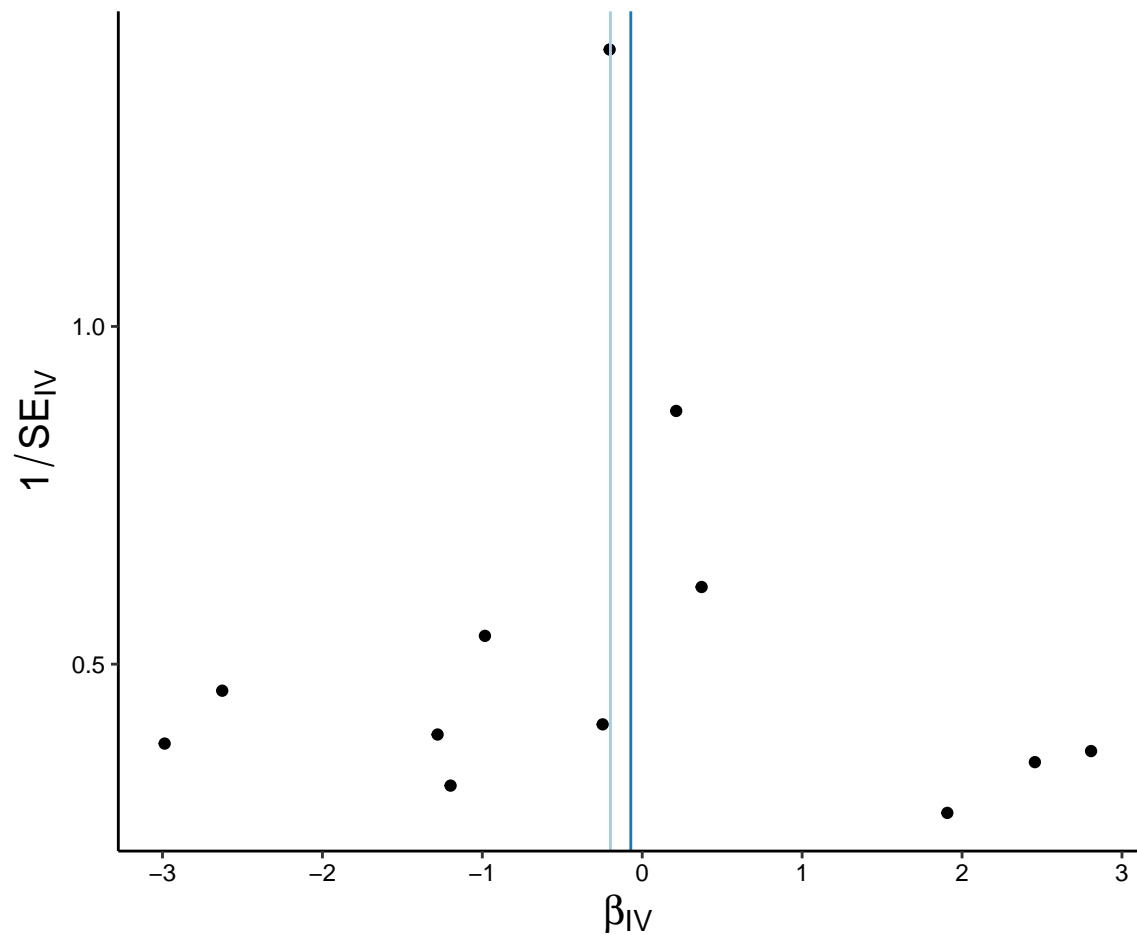

Supplement: Supplementary file 2 — Supporting Information 2 Figure S1: Funnel plots visualizing the heterogeneity of the significant causal associations identified in the main analysis. Figure S2: Leave‐one‐out sensitivity analysis plots for the significant causal associations. [file JDR-2026-9935331-s002.zip › Figure S1/Fig.S1C.pdf]

# MR Method

Inverse variance weighted

MR Egger

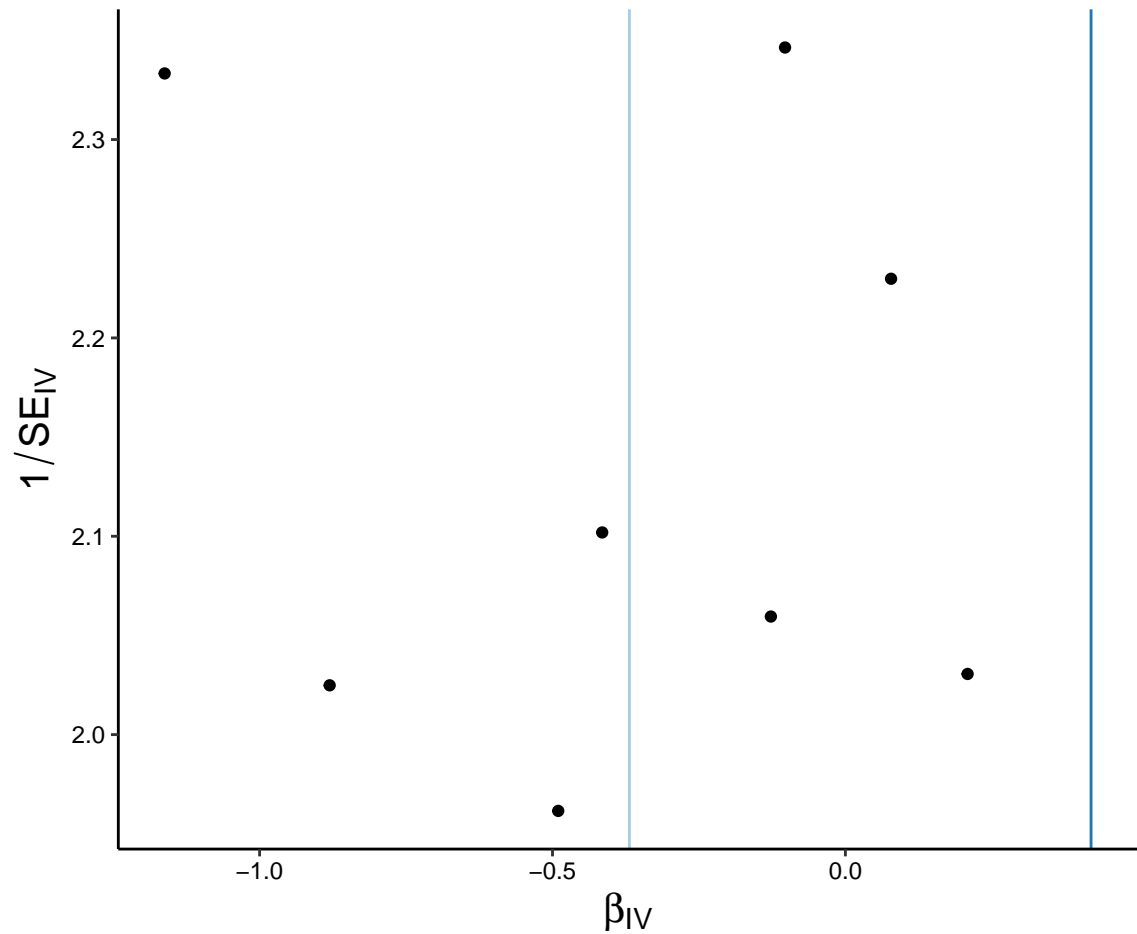

Supplement: Supplementary file 2 — Supporting Information 2 Figure S1: Funnel plots visualizing the heterogeneity of the significant causal associations identified in the main analysis. Figure S2: Leave‐one‐out sensitivity analysis plots for the significant causal associations. [file JDR-2026-9935331-s002.zip › Figure S1/Fig.S1D.pdf]

# MR Method

Inverse variance weighted

MR Egger

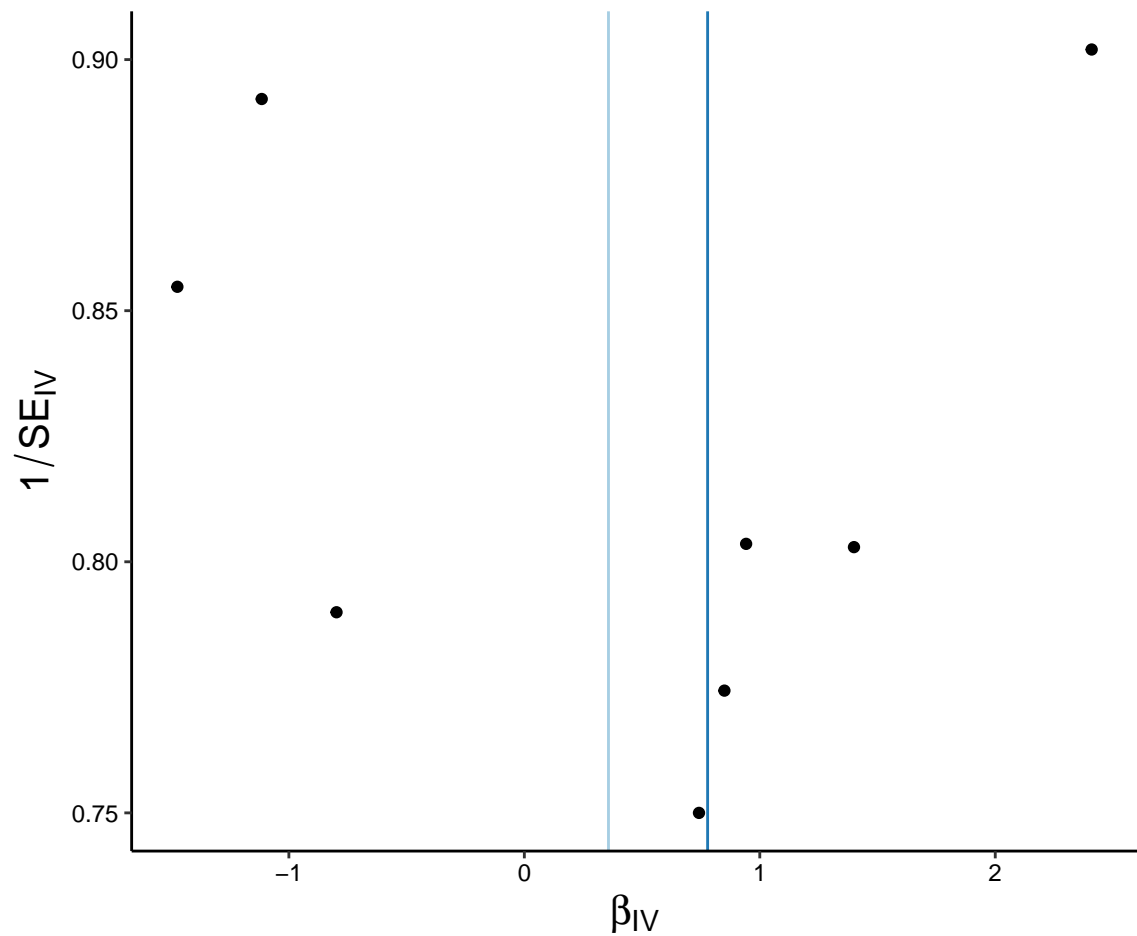

Supplement: Supplementary file 2 — Supporting Information 2 Figure S1: Funnel plots visualizing the heterogeneity of the significant causal associations identified in the main analysis. Figure S2: Leave‐one‐out sensitivity analysis plots for the significant causal associations. [file JDR-2026-9935331-s002.zip › Figure S1/Fig.S1E.pdf]

# MR Method

Inverse variance weighted

MR Egger

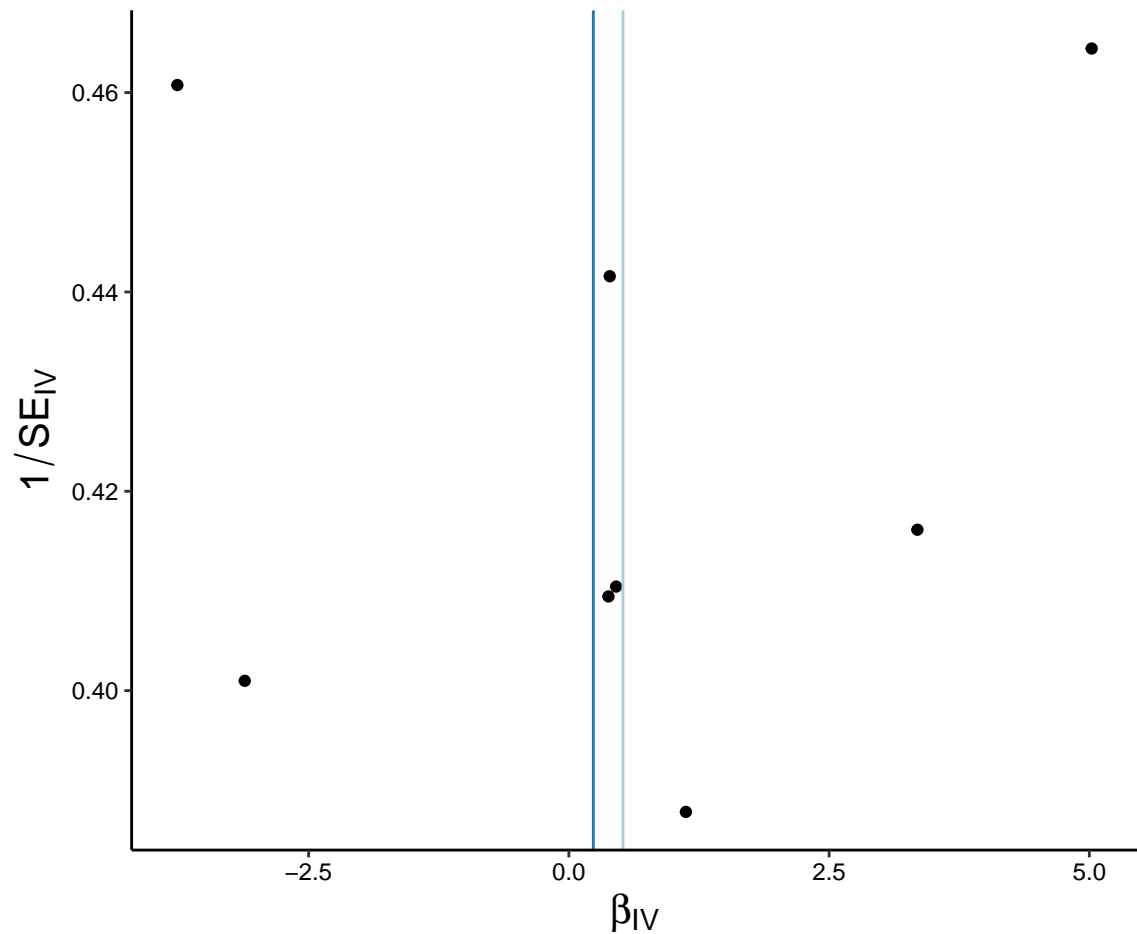

Supplement: Supplementary file 2 — Supporting Information 2 Figure S1: Funnel plots visualizing the heterogeneity of the significant causal associations identified in the main analysis. Figure S2: Leave‐one‐out sensitivity analysis plots for the significant causal associations. [file JDR-2026-9935331-s002.zip › Figure S1/Fig.S1F.pdf]

# MR Method

Inverse variance weighted

MR Egger

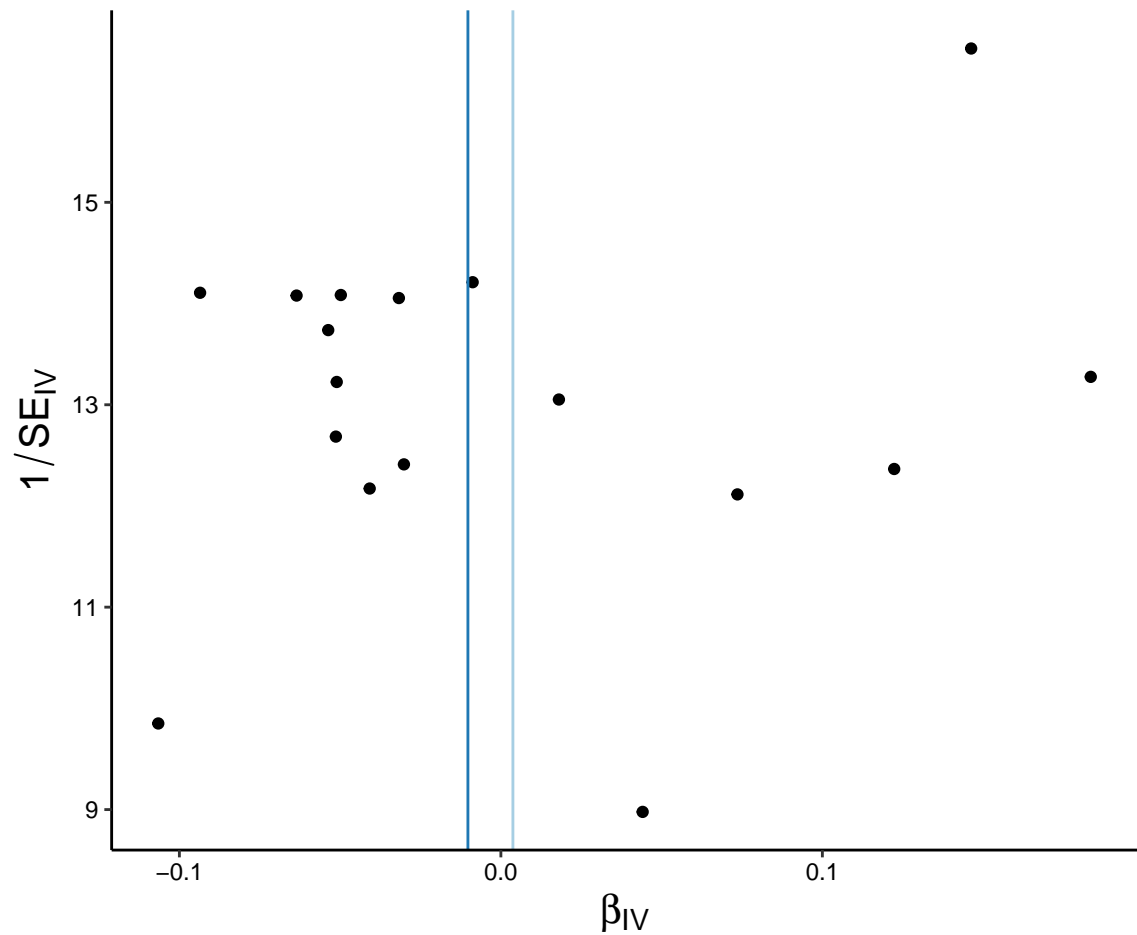

Supplement: Supplementary file 2 — Supporting Information 2 Figure S1: Funnel plots visualizing the heterogeneity of the significant causal associations identified in the main analysis. Figure S2: Leave‐one‐out sensitivity analysis plots for the significant causal associations. [file JDR-2026-9935331-s002.zip › Figure S1/Fig.S1G.pdf]

# MR Method

Inverse variance weighted

MR Egger

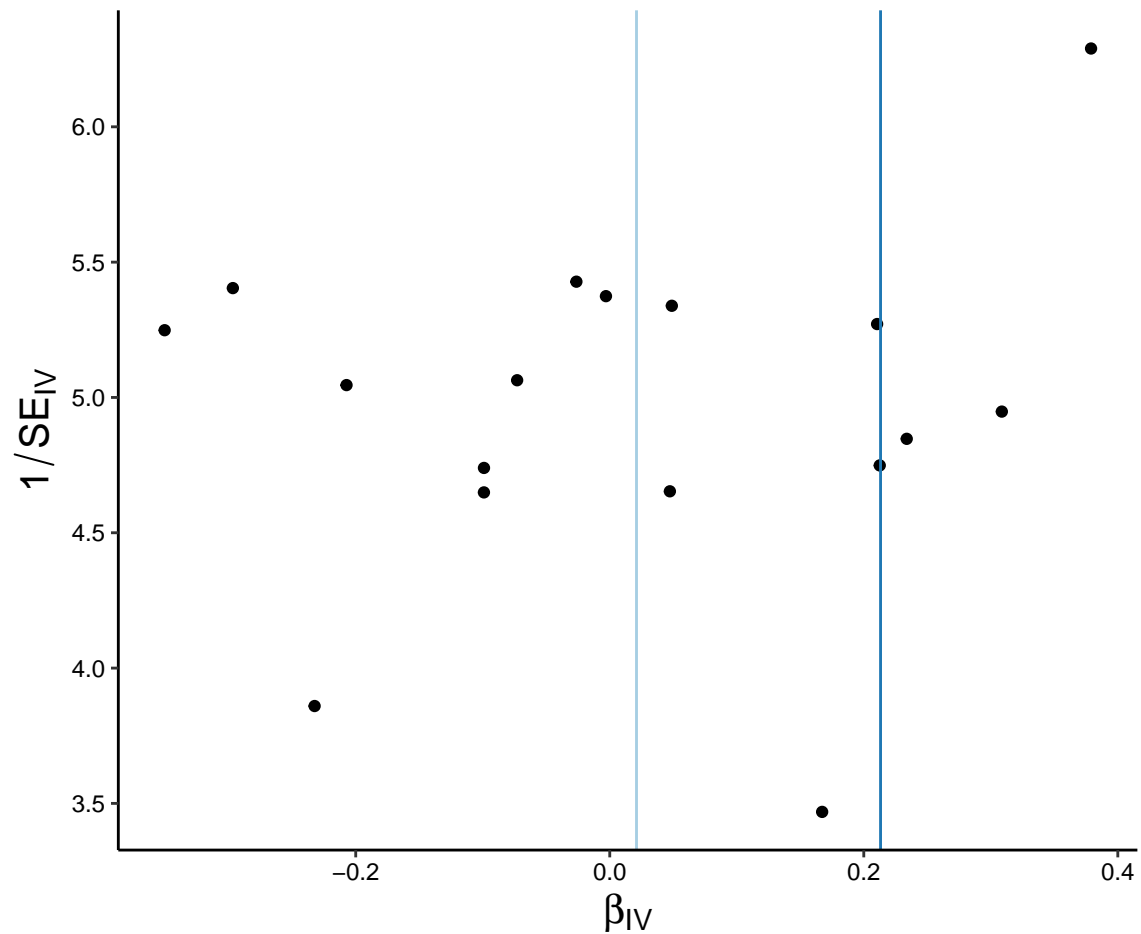

Supplement: Supplementary file 2 — Supporting Information 2 Figure S1: Funnel plots visualizing the heterogeneity of the significant causal associations identified in the main analysis. Figure S2: Leave‐one‐out sensitivity analysis plots for the significant causal associations. [file JDR-2026-9935331-s002.zip › Figure S1/Fig.S1H.pdf]

# MR Method

Inverse variance weighted

MR Egger

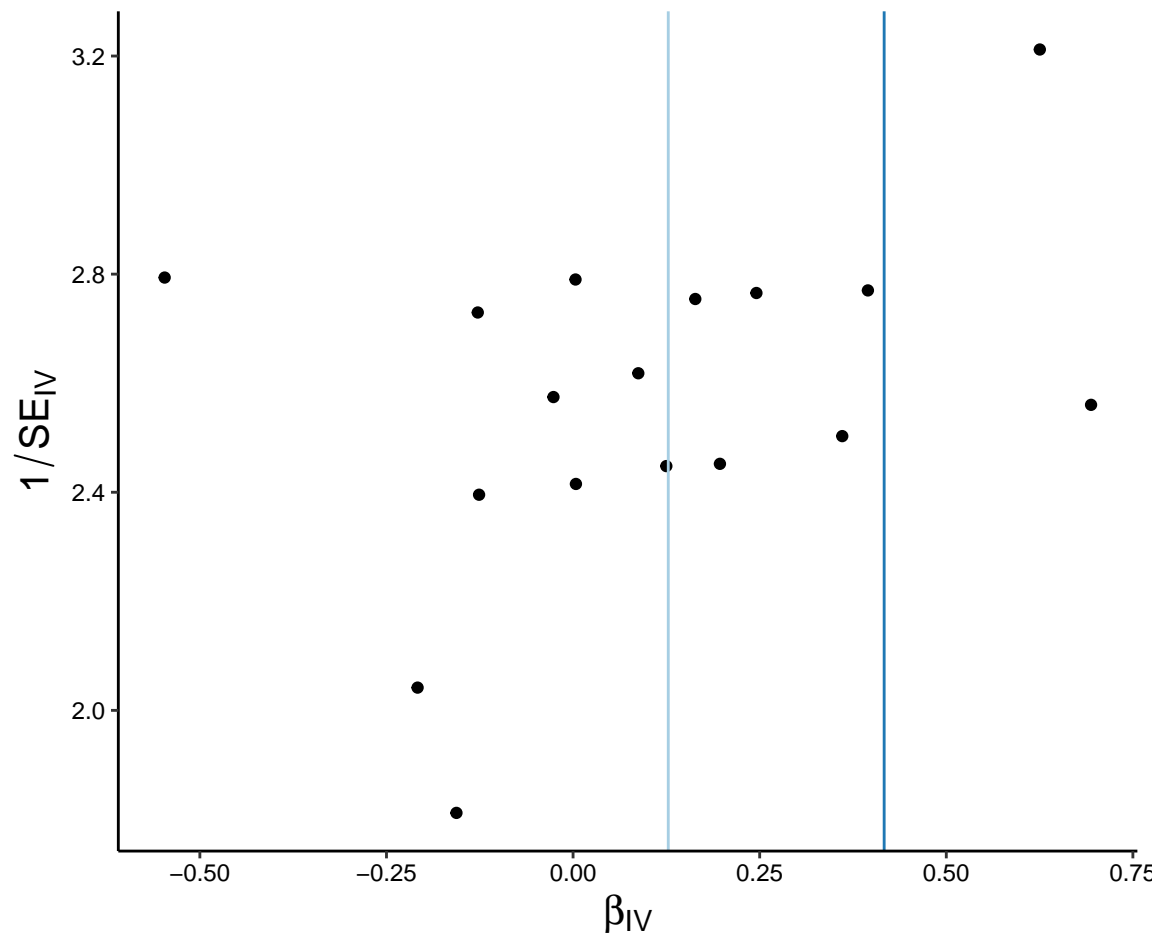

Supplement: Supplementary file 2 — Supporting Information 2 Figure S1: Funnel plots visualizing the heterogeneity of the significant causal associations identified in the main analysis. Figure S2: Leave‐one‐out sensitivity analysis plots for the significant causal associations. [file JDR-2026-9935331-s002.zip › Figure S1/Fig.S1I.pdf]

# MR Method

Inverse variance weighted

MR Egger

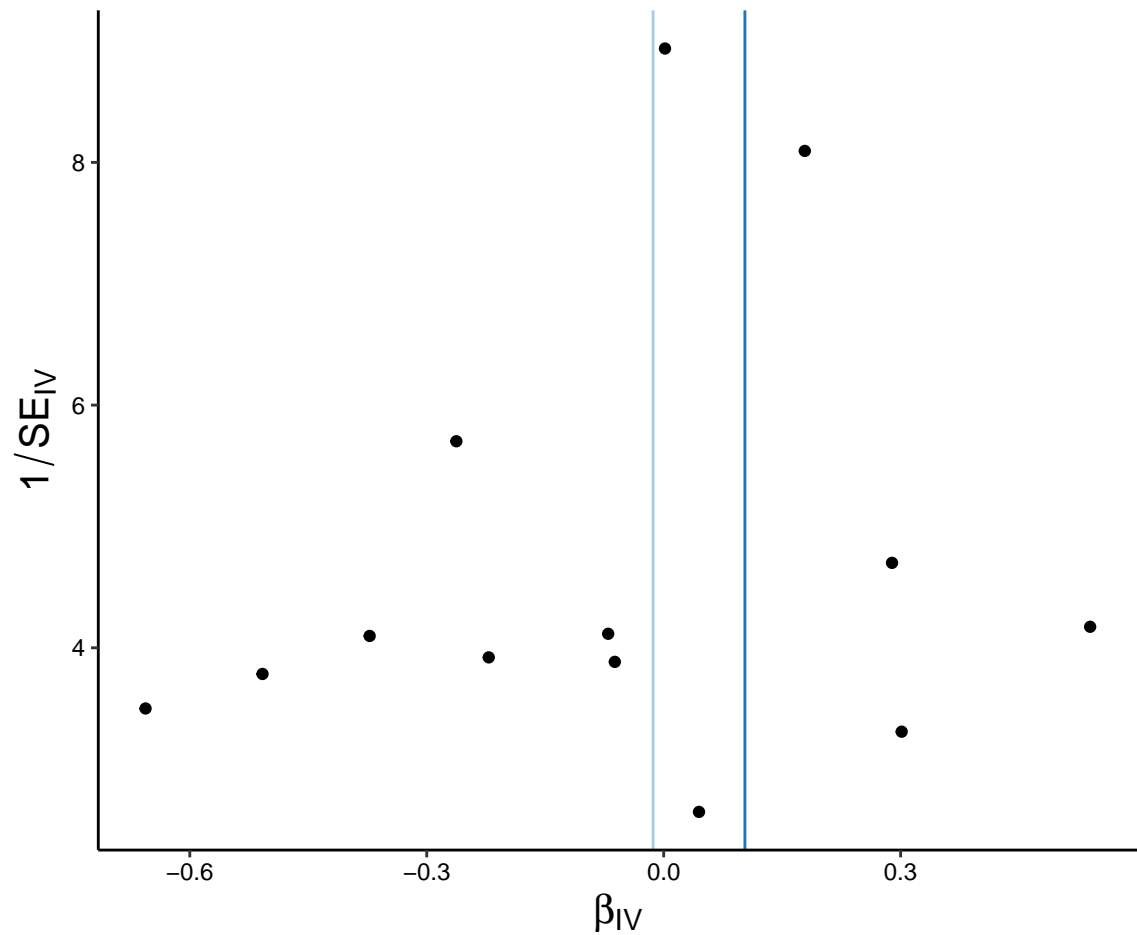

Supplement: Supplementary file 2 — Supporting Information 2 Figure S1: Funnel plots visualizing the heterogeneity of the significant causal associations identified in the main analysis. Figure S2: Leave‐one‐out sensitivity analysis plots for the significant causal associations. [file JDR-2026-9935331-s002.zip › Figure S1/Fig.S1J.pdf]

# MR Method

Inverse variance weighted

MR Egger

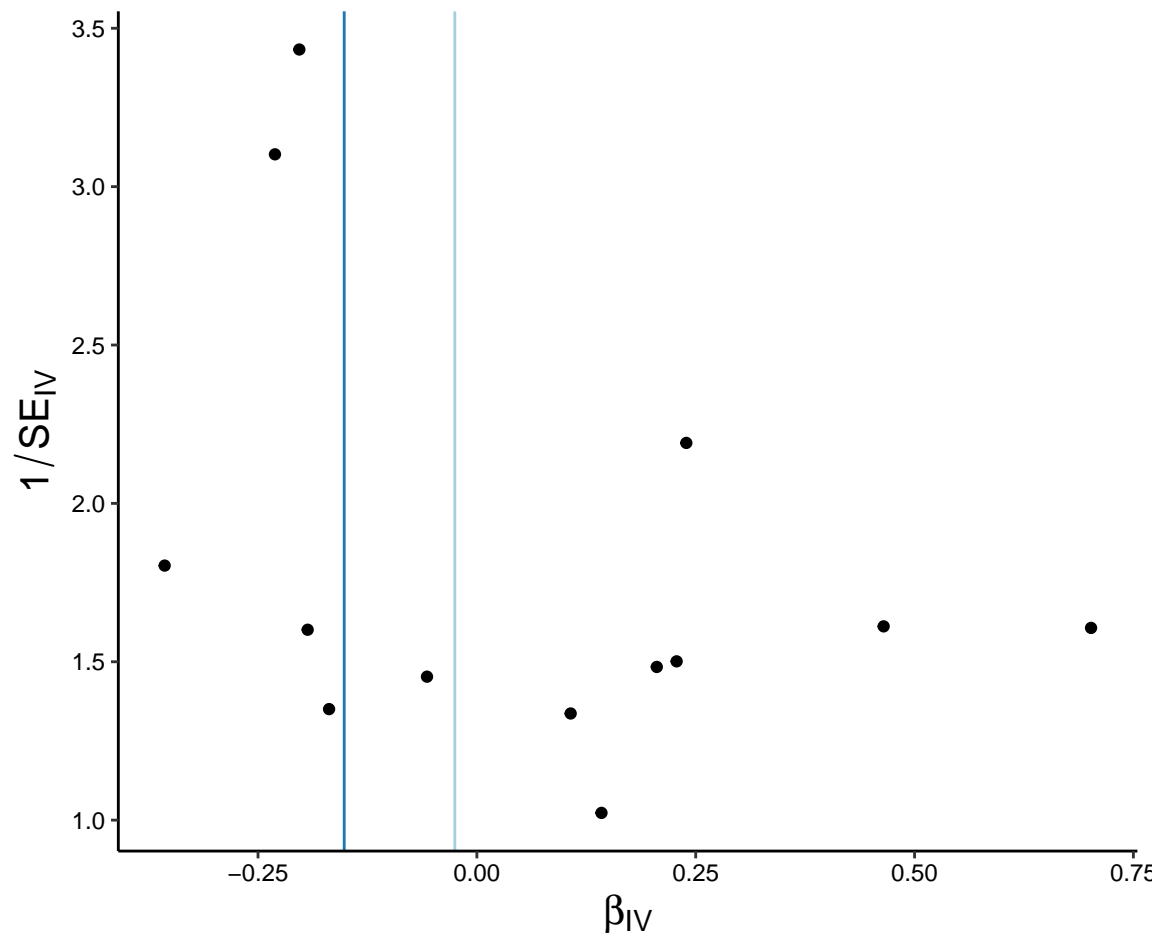

Supplement: Supplementary file 2 — Supporting Information 2 Figure S1: Funnel plots visualizing the heterogeneity of the significant causal associations identified in the main analysis. Figure S2: Leave‐one‐out sensitivity analysis plots for the significant causal associations. [file JDR-2026-9935331-s002.zip › Figure S1/Fig.S1K.pdf]

# MR Method

Inverse variance weighted

MR Egger

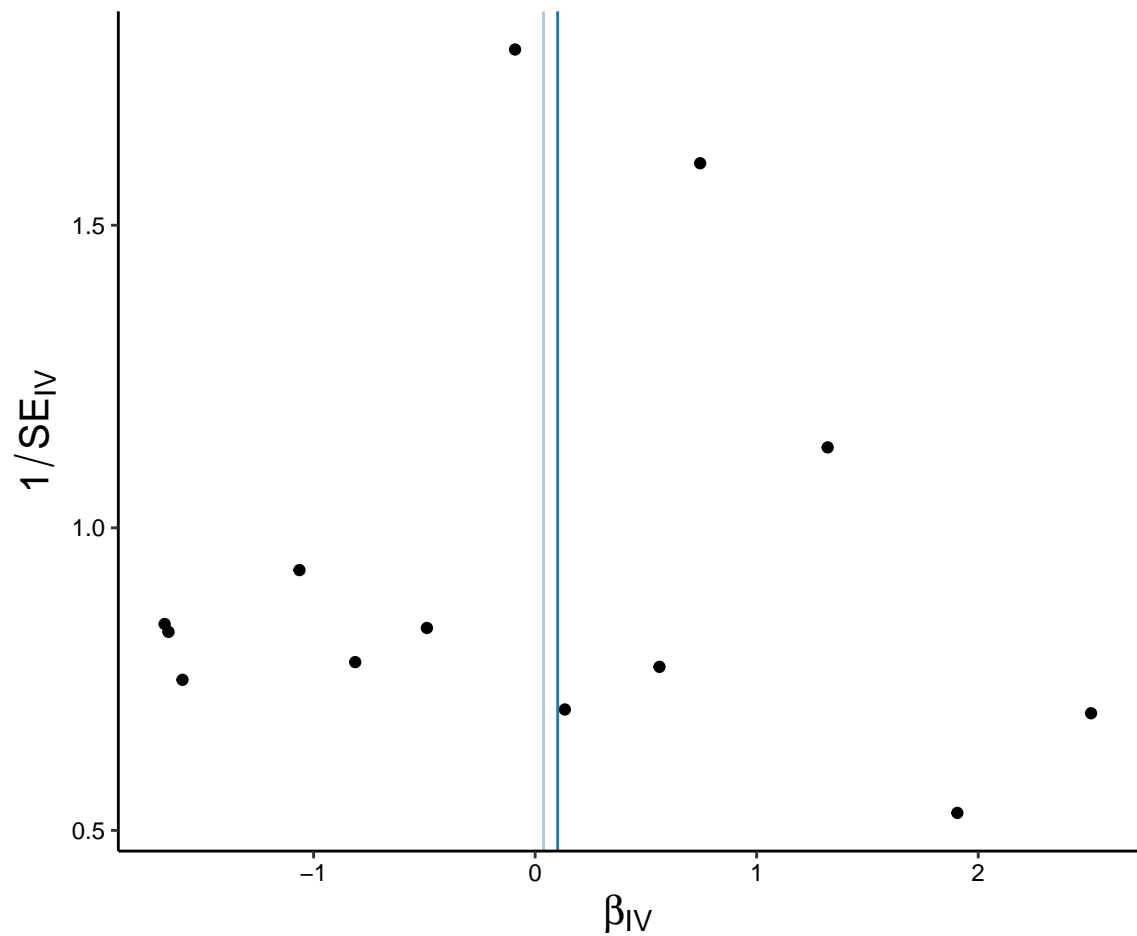

Supplement: Supplementary file 2 — Supporting Information 2 Figure S1: Funnel plots visualizing the heterogeneity of the significant causal associations identified in the main analysis. Figure S2: Leave‐one‐out sensitivity analysis plots for the significant causal associations. [file JDR-2026-9935331-s002.zip › Figure S1/Fig.S1L.pdf]

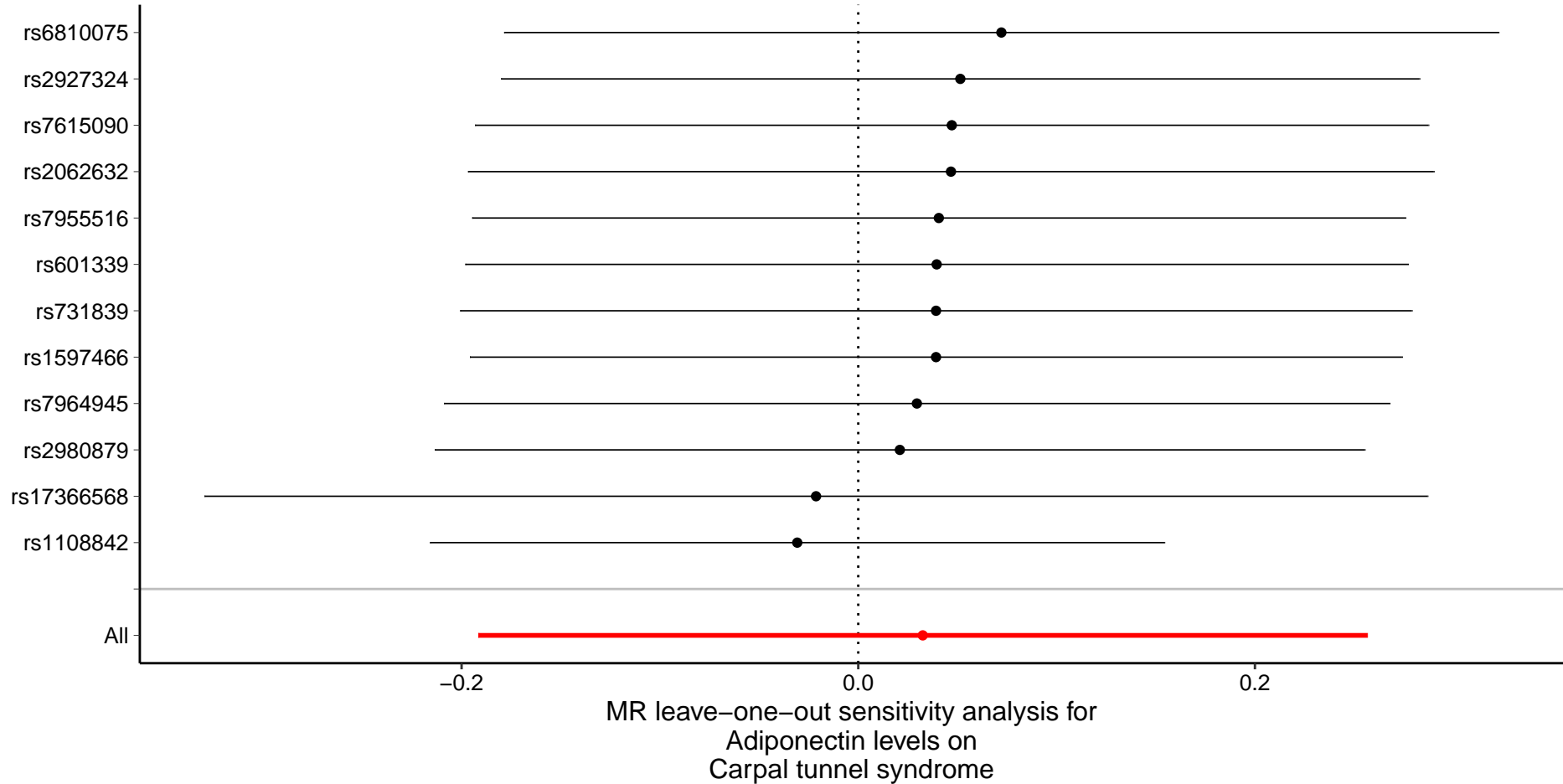

Supplement: Supplementary file 2 — Supporting Information 2 Figure S1: Funnel plots visualizing the heterogeneity of the significant causal associations identified in the main analysis. Figure S2: Leave‐one‐out sensitivity analysis plots for the significant causal associations. [file JDR-2026-9935331-s002.zip › Figure S2/Fig.S2A.pdf]

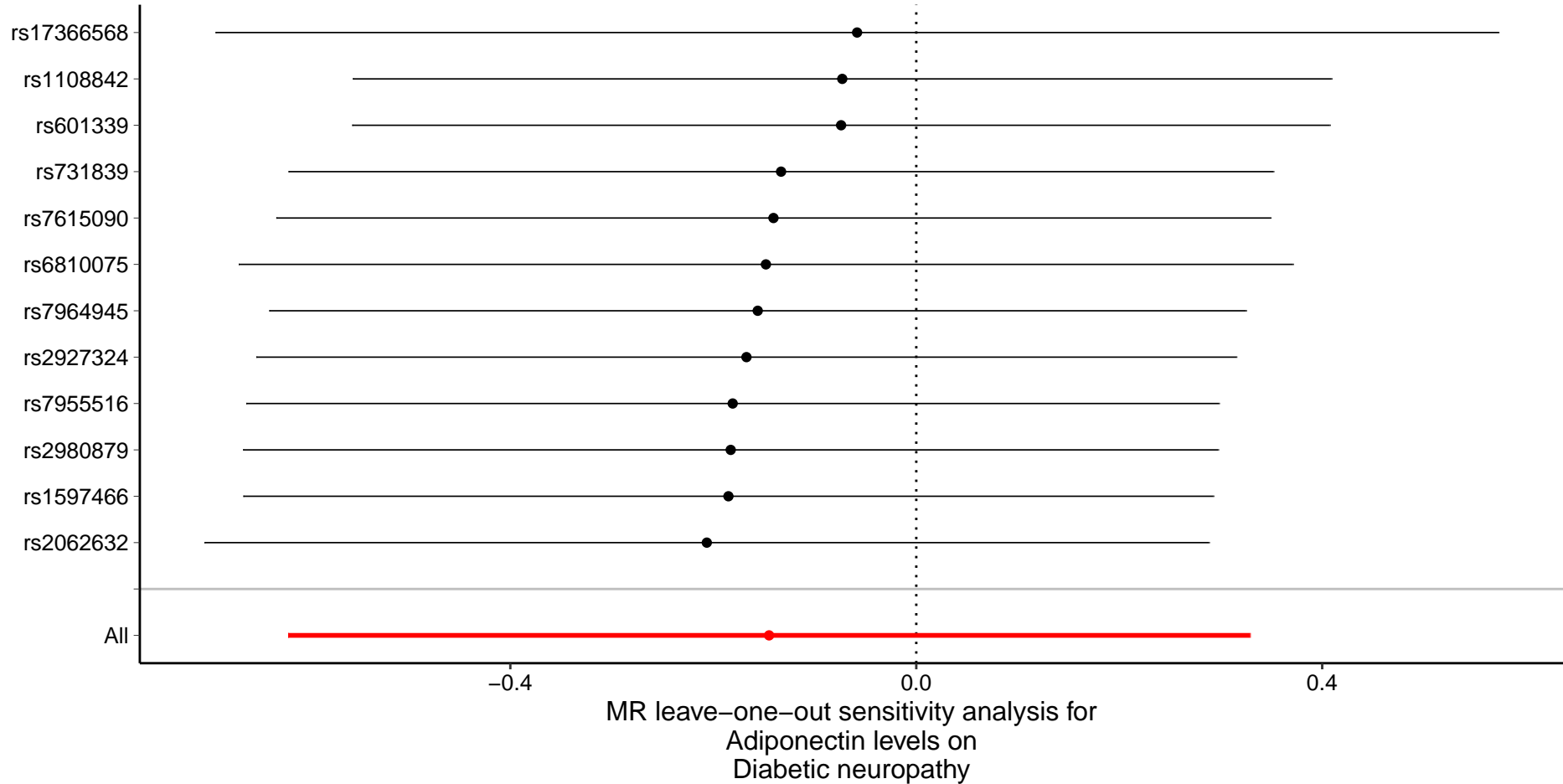

Supplement: Supplementary file 2 — Supporting Information 2 Figure S1: Funnel plots visualizing the heterogeneity of the significant causal associations identified in the main analysis. Figure S2: Leave‐one‐out sensitivity analysis plots for the significant causal associations. [file JDR-2026-9935331-s002.zip › Figure S2/Fig.S2B.pdf]

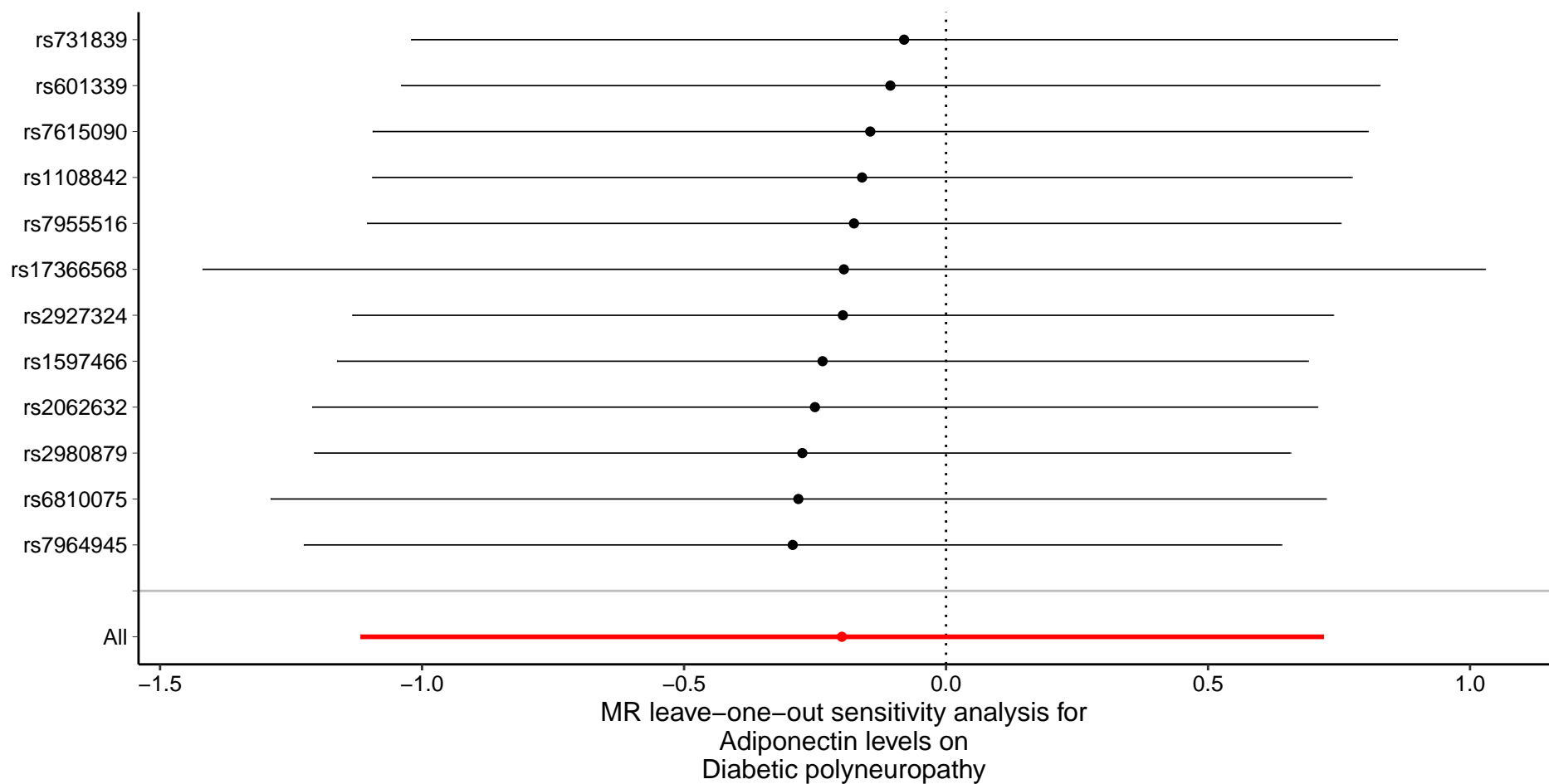

Supplement: Supplementary file 2 — Supporting Information 2 Figure S1: Funnel plots visualizing the heterogeneity of the significant causal associations identified in the main analysis. Figure S2: Leave‐one‐out sensitivity analysis plots for the significant causal associations. [file JDR-2026-9935331-s002.zip › Figure S2/Fig.S2C.pdf]

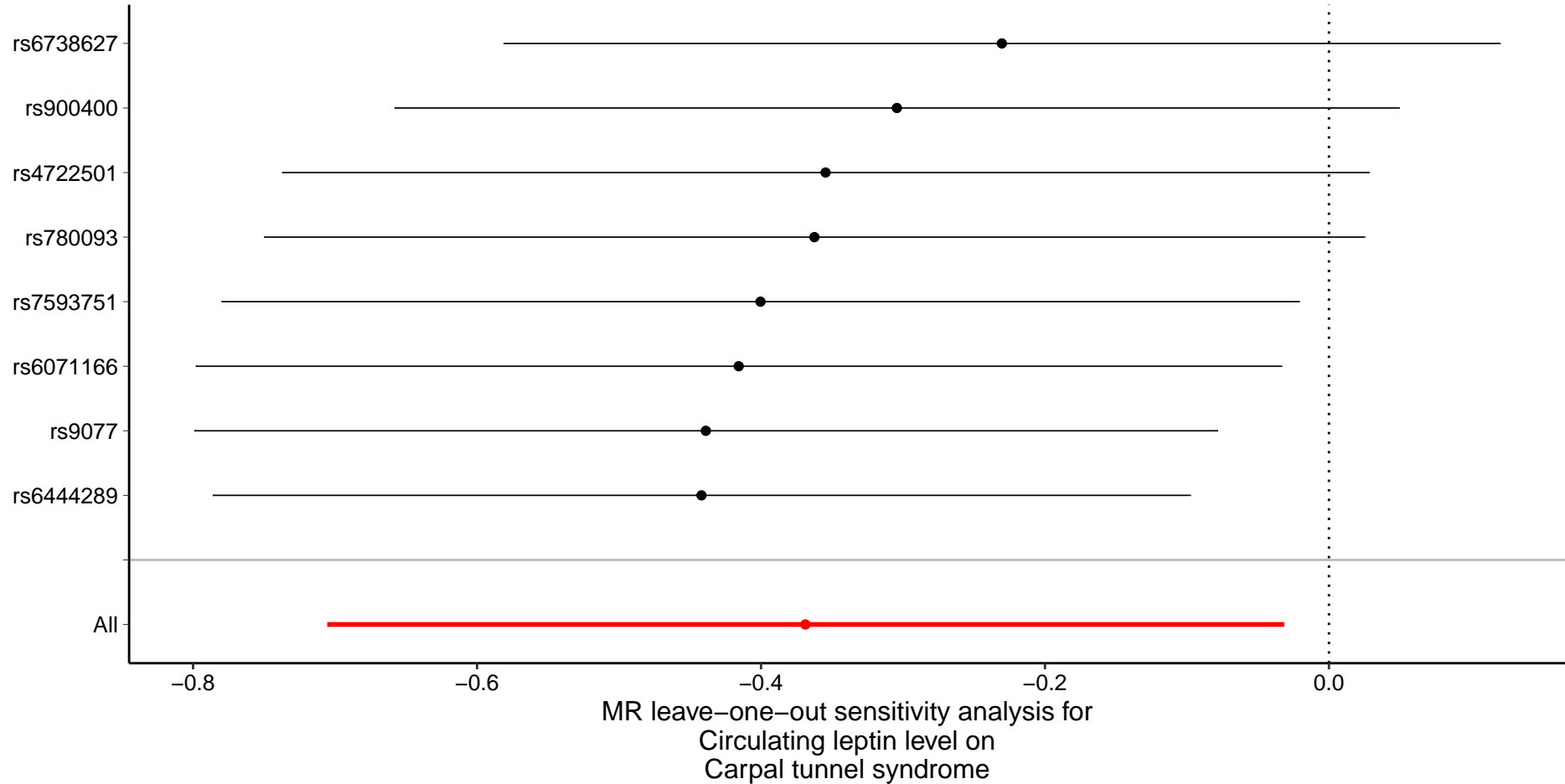

Supplement: Supplementary file 2 — Supporting Information 2 Figure S1: Funnel plots visualizing the heterogeneity of the significant causal associations identified in the main analysis. Figure S2: Leave‐one‐out sensitivity analysis plots for the significant causal associations. [file JDR-2026-9935331-s002.zip › Figure S2/Fig.S2D.pdf]

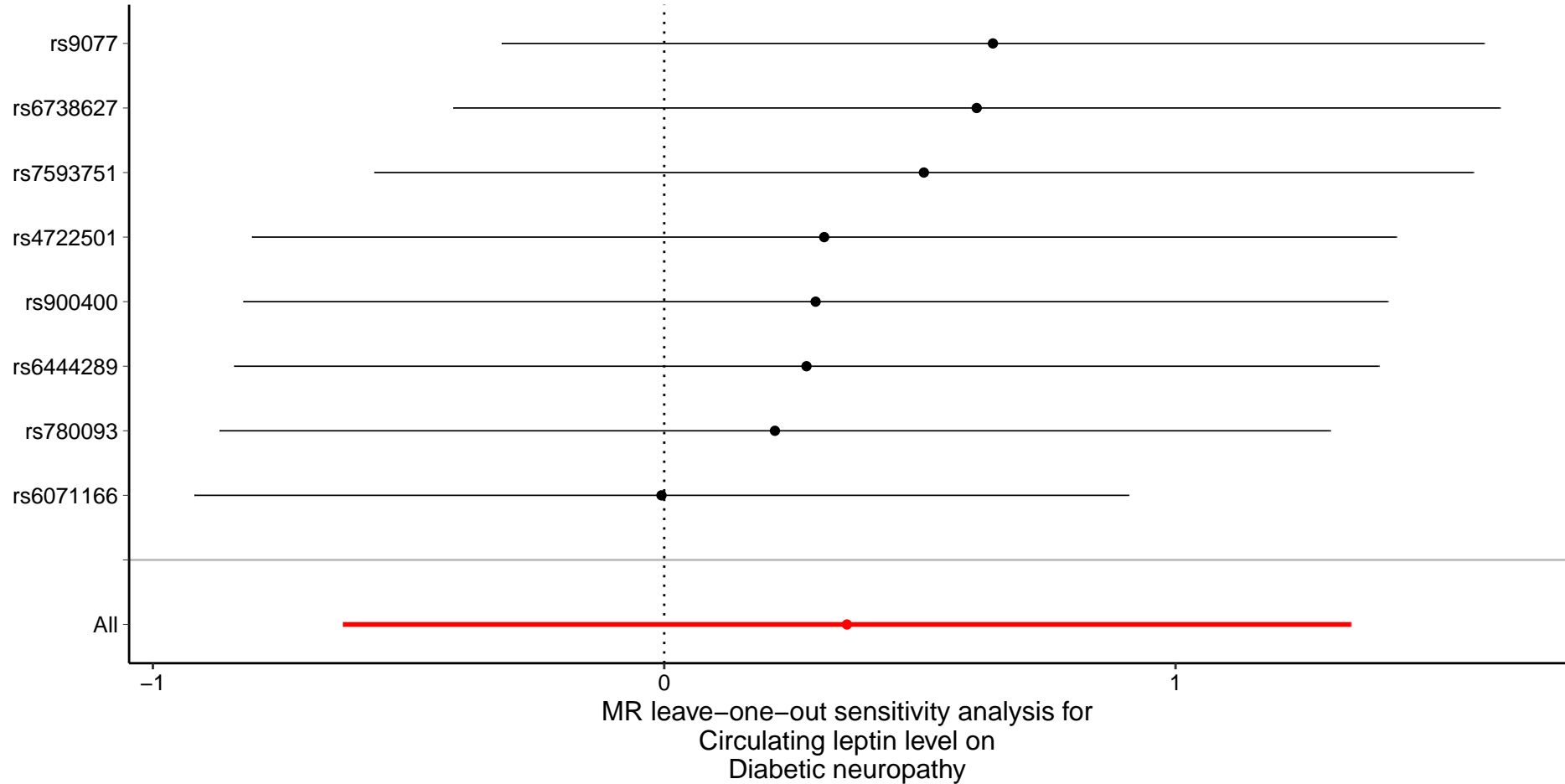

Supplement: Supplementary file 2 — Supporting Information 2 Figure S1: Funnel plots visualizing the heterogeneity of the significant causal associations identified in the main analysis. Figure S2: Leave‐one‐out sensitivity analysis plots for the significant causal associations. [file JDR-2026-9935331-s002.zip › Figure S2/Fig.S2E.pdf]

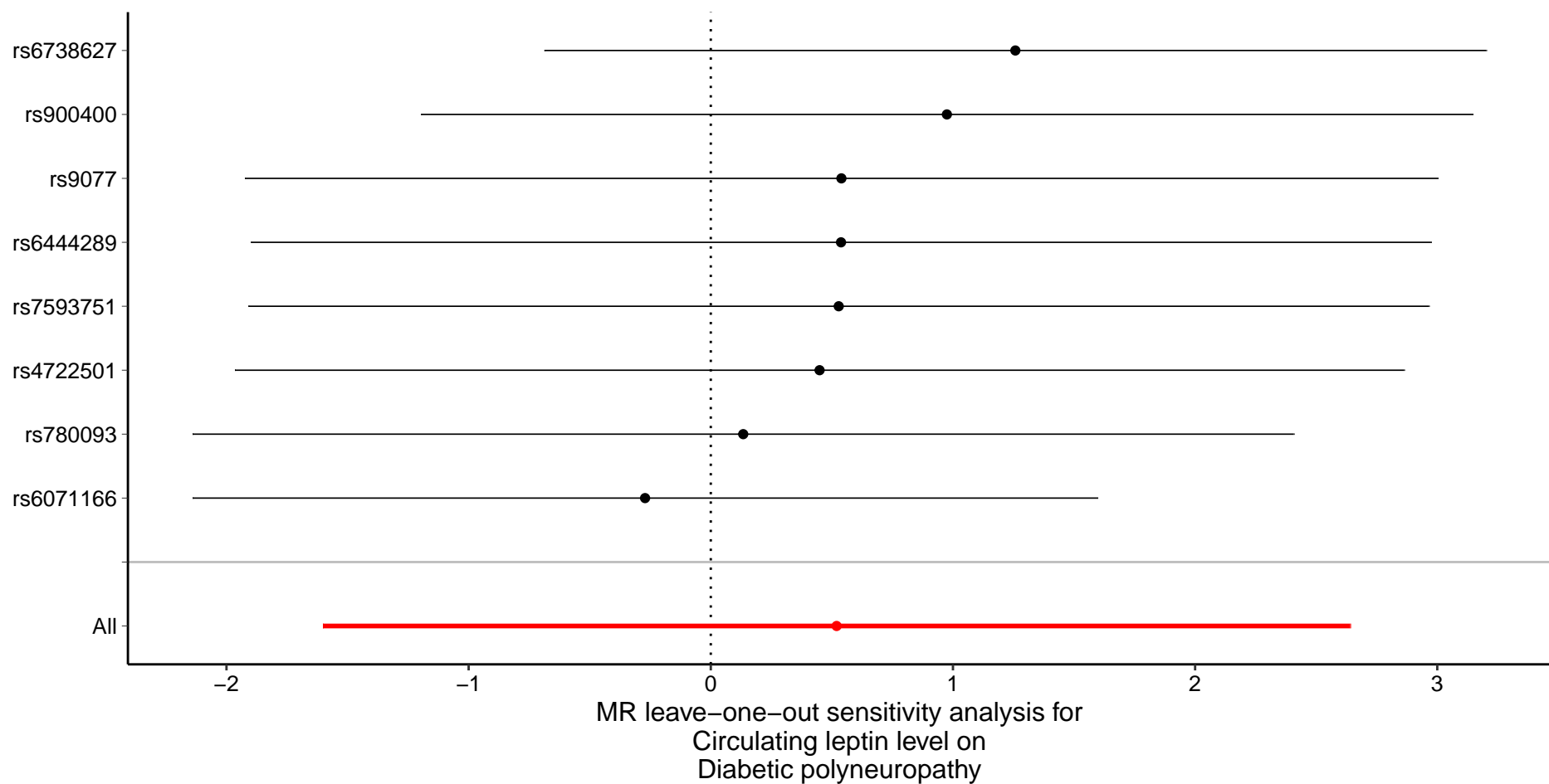

Supplement: Supplementary file 2 — Supporting Information 2 Figure S1: Funnel plots visualizing the heterogeneity of the significant causal associations identified in the main analysis. Figure S2: Leave‐one‐out sensitivity analysis plots for the significant causal associations. [file JDR-2026-9935331-s002.zip › Figure S2/Fig.S2F.pdf]

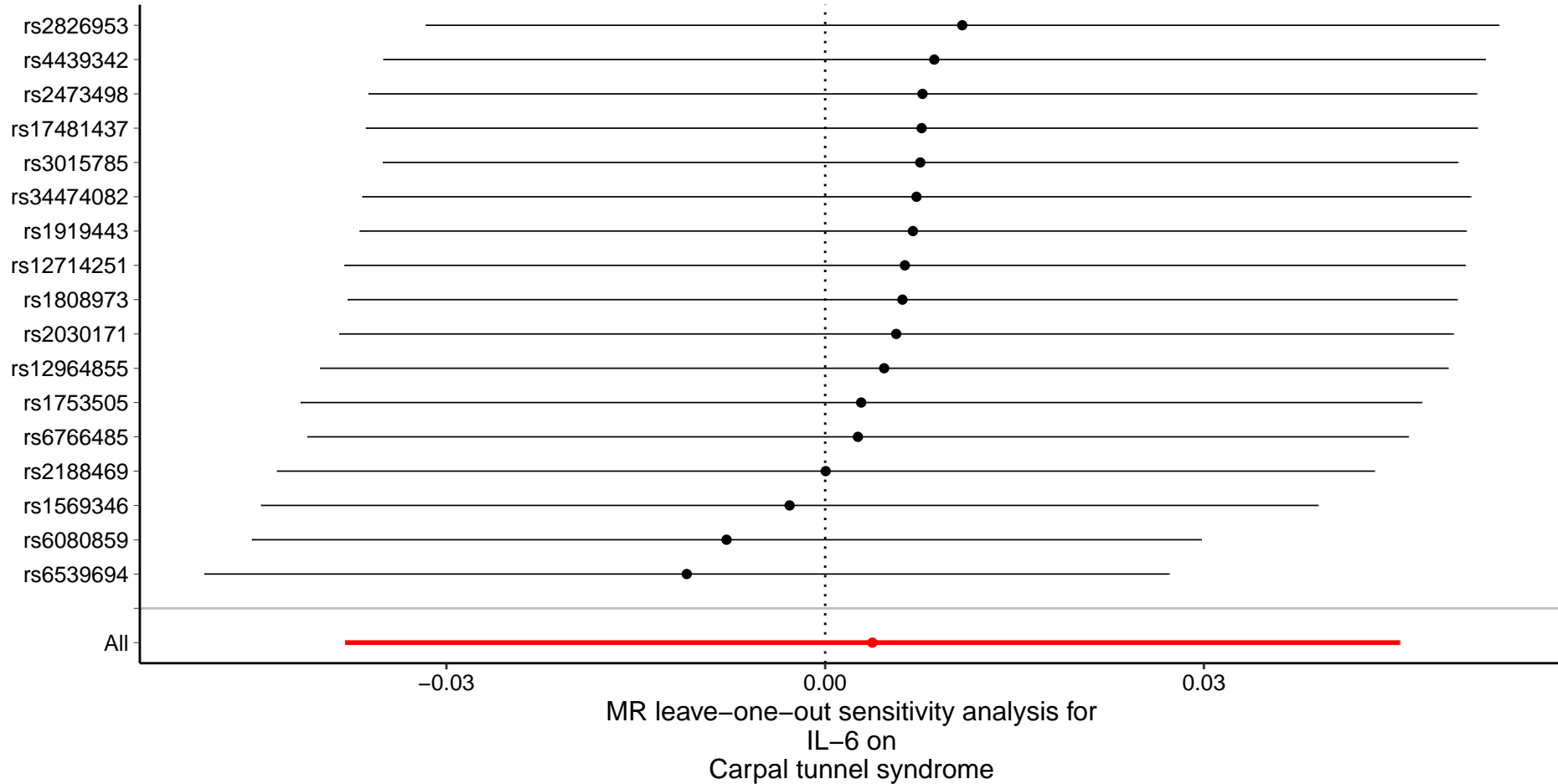

Supplement: Supplementary file 2 — Supporting Information 2 Figure S1: Funnel plots visualizing the heterogeneity of the significant causal associations identified in the main analysis. Figure S2: Leave‐one‐out sensitivity analysis plots for the significant causal associations. [file JDR-2026-9935331-s002.zip › Figure S2/Fig.S2G.pdf]

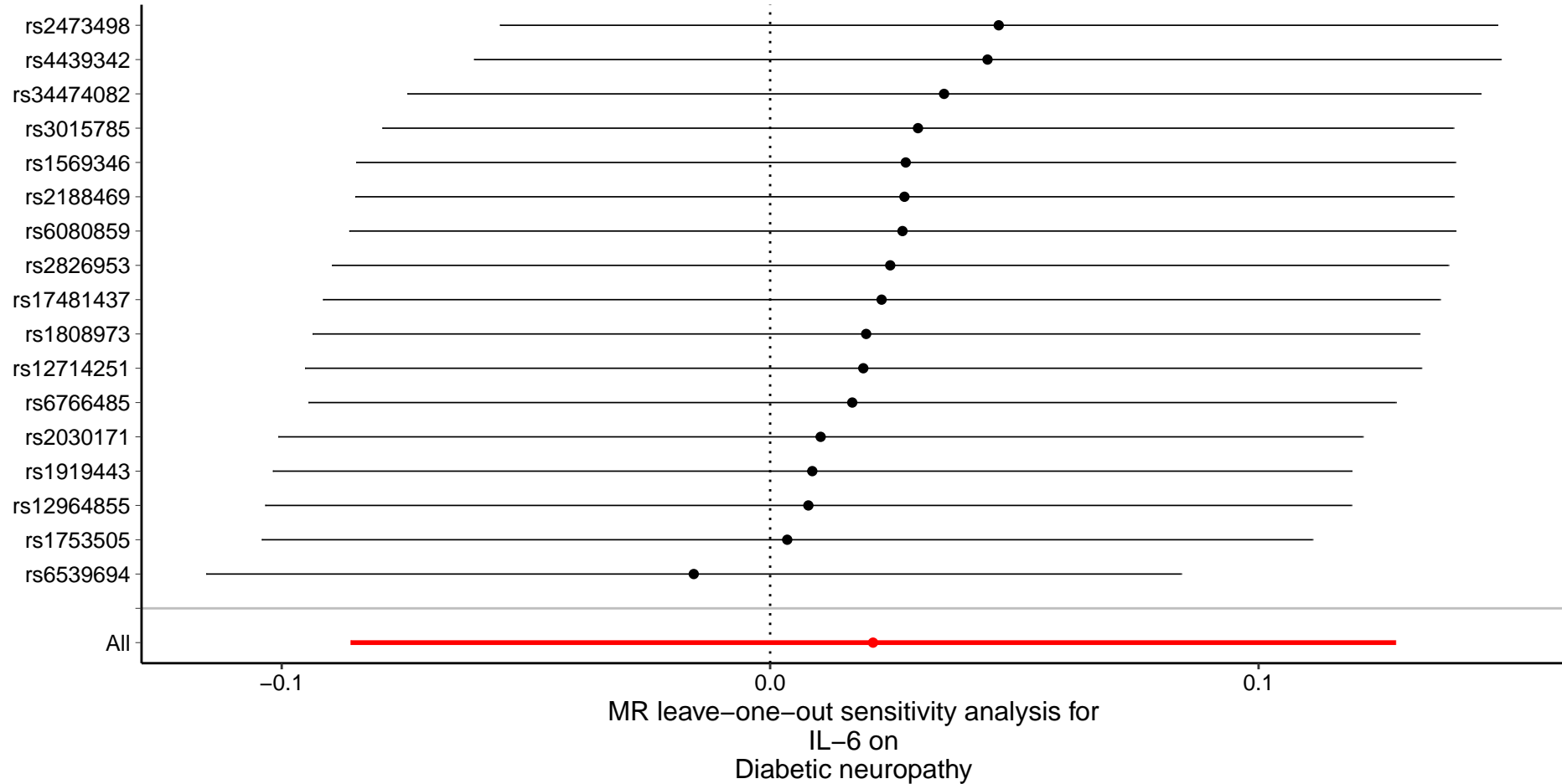

Supplement: Supplementary file 2 — Supporting Information 2 Figure S1: Funnel plots visualizing the heterogeneity of the significant causal associations identified in the main analysis. Figure S2: Leave‐one‐out sensitivity analysis plots for the significant causal associations. [file JDR-2026-9935331-s002.zip › Figure S2/Fig.S2H.pdf]

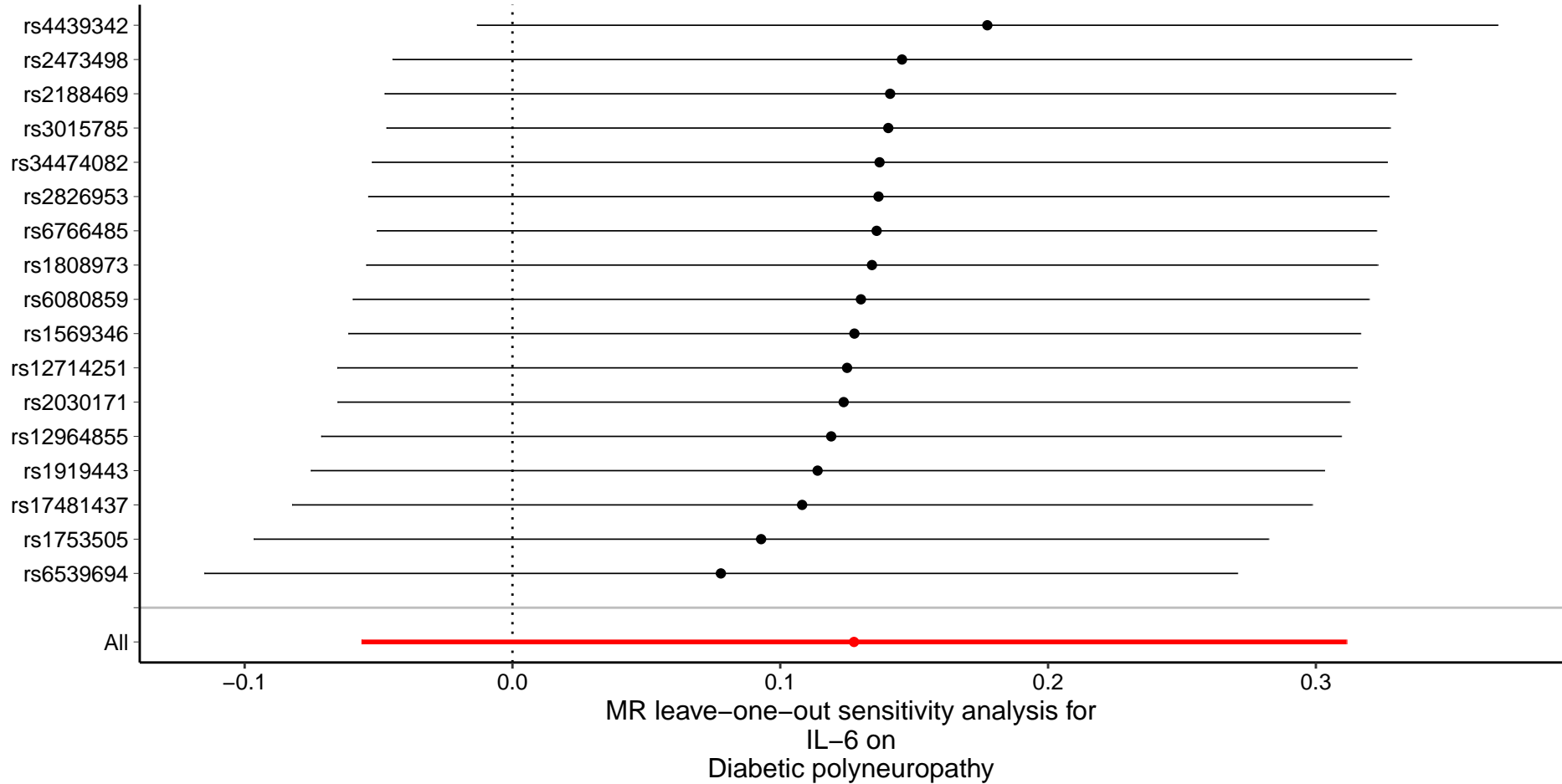

Supplement: Supplementary file 2 — Supporting Information 2 Figure S1: Funnel plots visualizing the heterogeneity of the significant causal associations identified in the main analysis. Figure S2: Leave‐one‐out sensitivity analysis plots for the significant causal associations. [file JDR-2026-9935331-s002.zip › Figure S2/Fig.S2I.pdf]

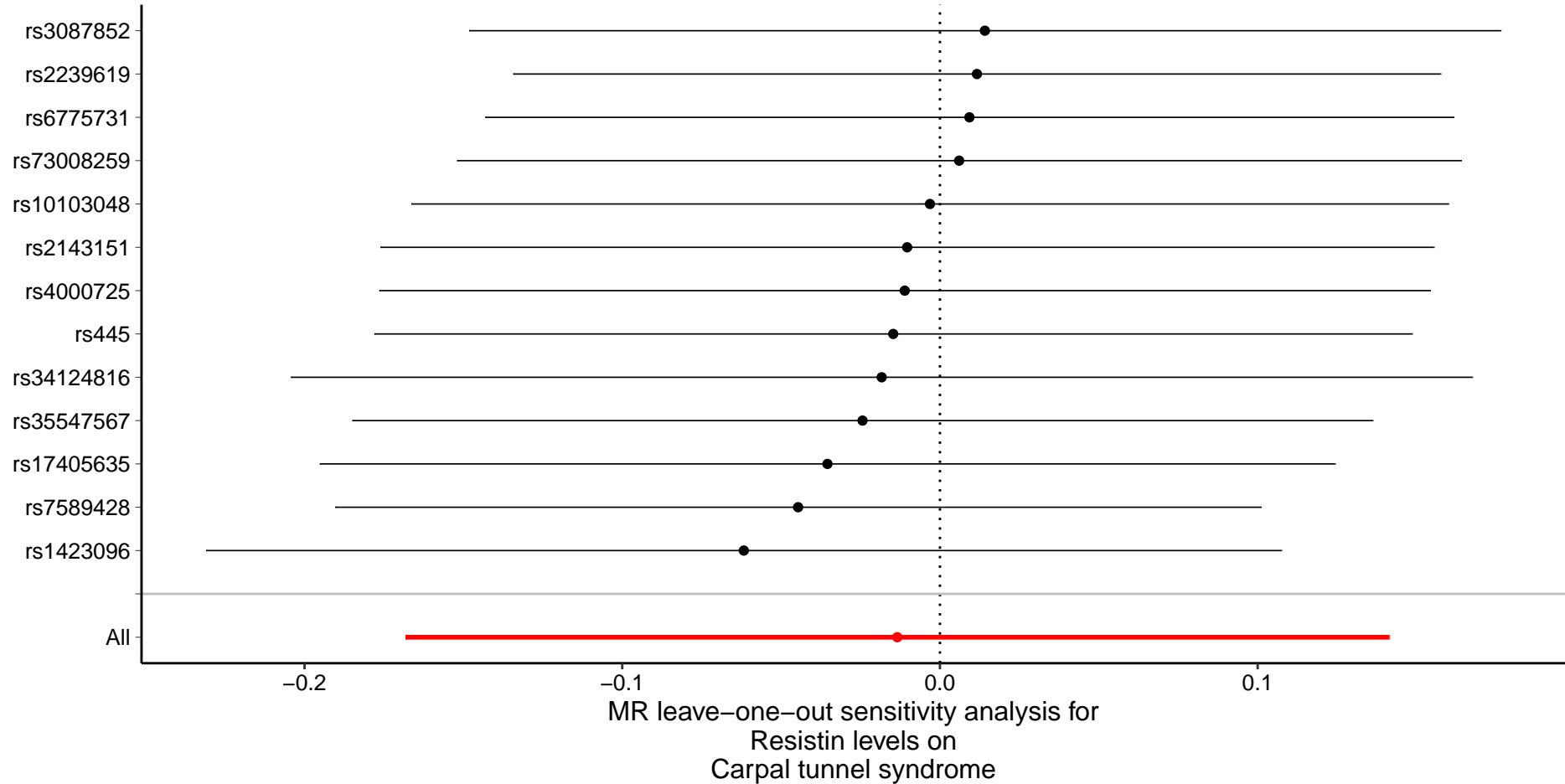

Supplement: Supplementary file 2 — Supporting Information 2 Figure S1: Funnel plots visualizing the heterogeneity of the significant causal associations identified in the main analysis. Figure S2: Leave‐one‐out sensitivity analysis plots for the significant causal associations. [file JDR-2026-9935331-s002.zip › Figure S2/Fig.S2J.pdf]

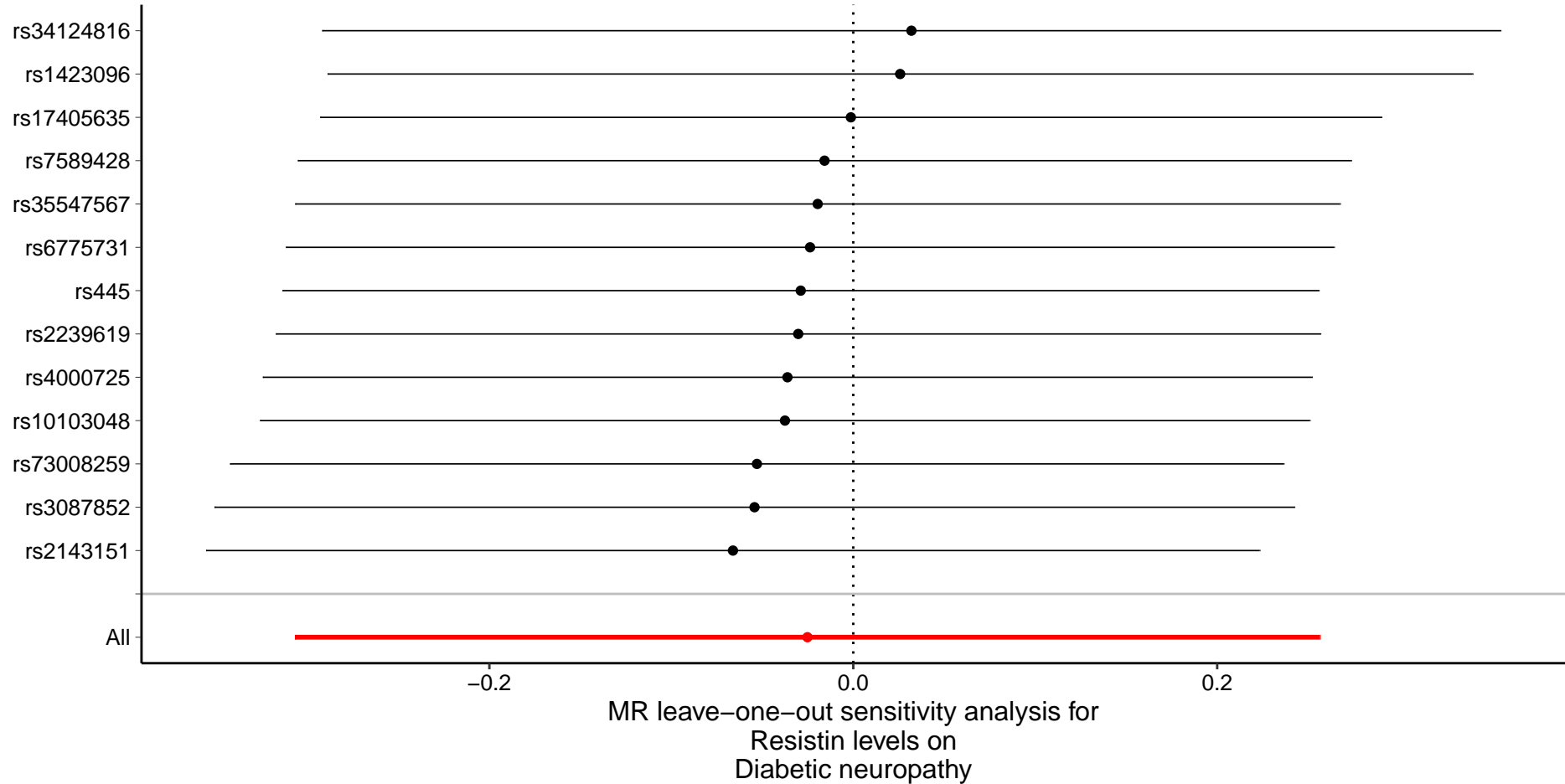

Supplement: Supplementary file 2 — Supporting Information 2 Figure S1: Funnel plots visualizing the heterogeneity of the significant causal associations identified in the main analysis. Figure S2: Leave‐one‐out sensitivity analysis plots for the significant causal associations. [file JDR-2026-9935331-s002.zip › Figure S2/Fig.S2K.pdf]

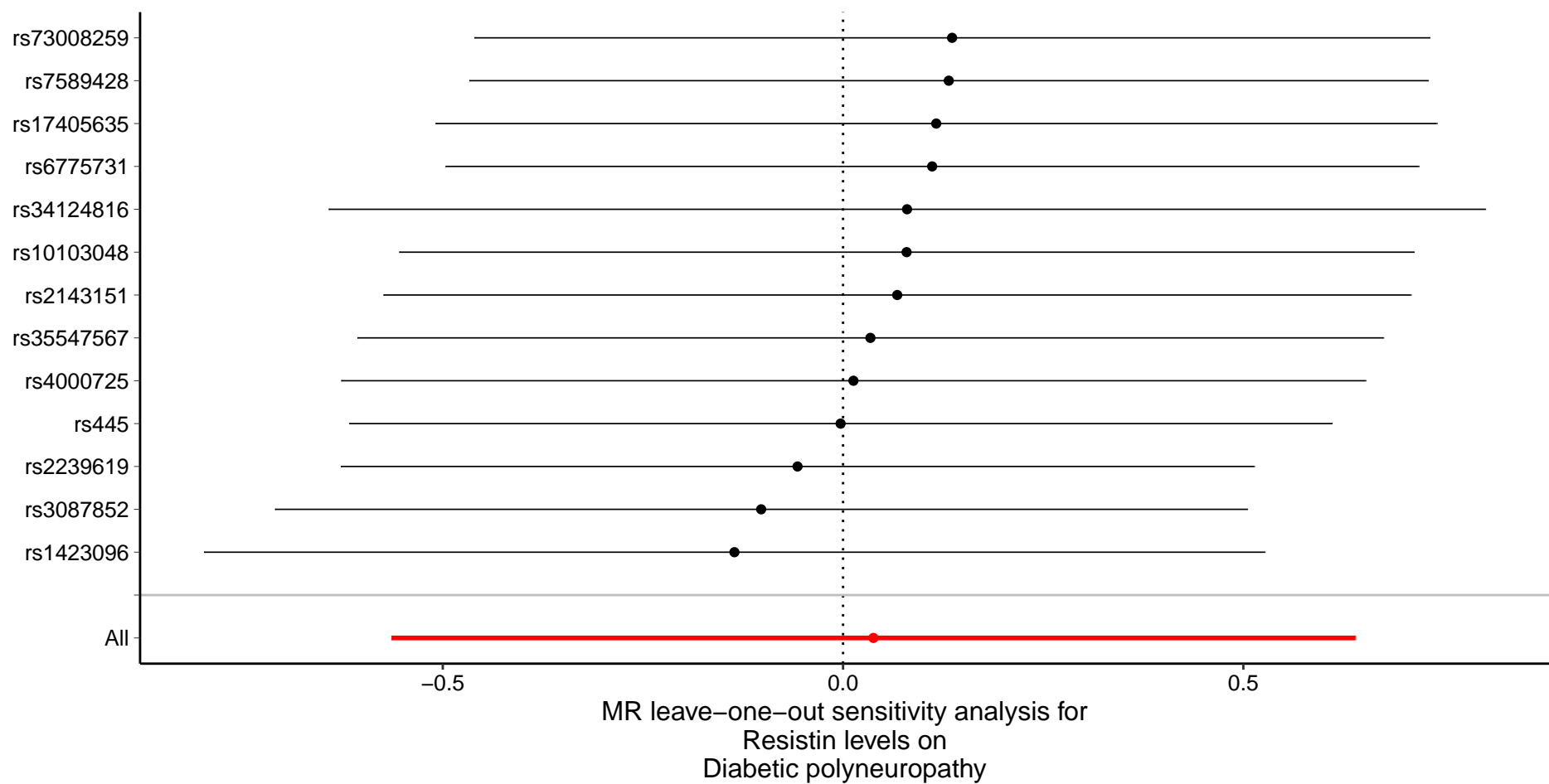

Supplement: Supplementary file 2 — Supporting Information 2 Figure S1: Funnel plots visualizing the heterogeneity of the significant causal associations identified in the main analysis. Figure S2: Leave‐one‐out sensitivity analysis plots for the significant causal associations. [file JDR-2026-9935331-s002.zip › Figure S2/Fig.S2L.pdf]
